# Capacity of countries to reduce biological invasions

**DOI:** 10.1007/s11625-022-01166-3

**Published:** 2022-07-20

**Authors:** Guillaume Latombe, Hanno Seebens, Bernd Lenzner, Franck Courchamp, Stefan Dullinger, Marina Golivets, Ingolf Kühn, Brian Leung, Núria Roura-Pascual, Emma Cebrian, Wayne Dawson, Christophe Diagne, Jonathan M. Jeschke, Cristian Pérez-Granados, Dietmar Moser, Anna Turbelin, Piero Visconti, Franz Essl

**Affiliations:** 1 BioInvasions, Global Change, Macroecology-Group, Department of Botany and Biodiversity Research, University of Vienna, Rennweg 14, 1030 Vienna, Austria; 2 Institute of Ecology and Evolution, The University of Edinburgh, King’s Buildings, Edinburgh, EH9 3FL UK; 3 Senckenberg Biodiversity and Climate Research Centre, Senckenberganlage 25, 60325 Frankfurt, Germany; 4 Université Paris-Saclay, CNRS, AgroParisTech, Ecologie Systématique Evolution, 91405 Orsay, France; 5 Helmholtz Centre for Environmental Research-UFZ, Theodor-Lieser-Str. 4, 06120 Halle, Germany; 6 Geobotany and Botanical Garden, Martin Luther University Halle-Wittenberg, 06099 Halle, Germany; 7 German Centre for Integrative Biodiversity Research (iDiv) Halle-Jena-Leipzig, Puschstraße 4, 04103 Leipzig, Germany; 8 Department of Biology, McGill University, Montreal, QC H3A 1B1 Canada; 9 Departament de Ciències Ambientals, Facultat de Ciències, Universitat de Girona, 17003 Girona, Catalonia Spain; 10 Centre d’Estudis Avançats de Blanes-CSIC, 17003 Girona, Spain; 11 GRMAR, Institute of Aquatic Ecology, University of Girona, 17003 Girona, Spain; 12 Department of Biosciences, Durham University, South Road, Durham, DH1 3LE UK; 13 CBGP, Univ Montpellier, CIRAD, INRAE, Institut Agro, IRD, Montpellier, France; 14 Institute of Biology, Freie Universität Berlin, 14195 Berlin, Germany; 15 Leibniz Institute of Freshwater Ecology and Inland Fisheries (IGB), 12587 Berlin, Germany; 16 Berlin-Brandenburg Institute of Advanced Biodiversity Research (BBIB), 14195 Berlin, Germany; 17 Ecology Department, Universidad de Alicante, 03080 Alicante, Spain; 18 Biodiversity, Ecology and Conservation Group, International Institute for Applied System Analyses, 2361 Laxenburg, Austria

**Keywords:** Environmental performance, Established alien species, Governance, Innovation, Lifestyle, Scenarios, Trade

## Abstract

The extent and impacts of biological invasions on biodiversity are largely shaped by an array of socio-economic and environmental factors, which exhibit high variation among countries. Yet, a global analysis of how these factors vary across countries is currently lacking. Here, we investigate how five broad, country-specific socio-economic and environmental indices (Governance, Trade, Environmental Performance, Lifestyle and Education, Innovation) explain country-level (1) established alien species (EAS) richness of eight taxonomic groups, and (2) proactive or reactive capacity to prevent and manage biological invasions and their impacts. These indices underpin many aspects of the invasion process, including the introduction, establishment, spread and management of alien species. They are also general enough to enable a global comparison across countries, and are therefore essential for defining future scenarios for biological invasions. Models including Trade, Governance, Lifestyle and Education, or a combination of these, best explained EAS richness across taxonomic groups and national proactive or reactive capacity. Historical (1996 or averaged over 1996–2015) levels of Governance and Trade better explained both EAS richness and the capacity of countries to manage invasions than more recent (2015) levels, revealing a historical legacy with important implications for the future of biological invasions. Using Governance and Trade to define a two-dimensional socio-economic space in which the position of a country captures its capacity to address issues of biological invasions, we identified four main clusters of countries in 2015. Most countries had an increase in Trade over the past 25 years, but trajectories were more geographically heterogeneous for Governance. Declines in levels of Governance are concerning as they may be responsible for larger levels of invasions in the future. By identifying the factors influencing EAS richness and the regions most susceptible to changes in these factors, our results provide novel insights to integrate biological invasions into scenarios of biodiversity change to better inform decision-making for policy and the management of biological invasions.

**Supplementary Information:**

The online version contains supplementary material available at 10.1007/s11625-022-01166-3.

## Introduction

The proliferation of alien species—i.e. species that are intentionally or unintentionally introduced by humans in regions beyond their native ranges—has become a signature of human-induced global environmental change. A substantial proportion of these species has become a permanent addition to regional biotas (established alien species—EAS hereafter), some of which are a leading cause of biodiversity decline (Bellard et al. 2016; Maxwell et al. 2016; Seidl et al. 2018) and adversely affect human livelihoods (Bradshaw et al. 2016; Paini et al. 2016; IPBES 2019; Diagne et al. 2021). In response to growing threats from biological invasions, many countries with high richness of alien species have expanded and implemented new legislations on alien species since the 1990s (Turbelin et al. 2017). Nevertheless, globally, the number of EAS has been steadily increasing in recent decades, and this trend does not show any sign of saturation (Seebens et al. 2017). Meanwhile, the current state and particularly the future trajectories of EAS impacts remain highly uncertain (Latombe et al. 2019; Essl et al. 2020b). Still, there is a distinct lack of consideration of the spread, establishment and management of alien species and their resulting impacts when developing long-term global biodiversity conservation frameworks and scenarios (Courchamp et al. 2017; Lenzner et al. 2019). To develop these frameworks and scenarios, a better understanding of global factors driving biological invasions is necessary.

Environmental and economic factors have been repeatedly linked to biological invasions at the global scale (Essl et al. 2015; Seebens et al. 2015; Dawson et al. 2017a; Sardain et al. 2019). For example, species have been deliberately released because of their perceived or realised economic benefits (Pringle 2005). Additionally, experts also consider political, social and technological factors to be important (Essl et al. 2020b; Roura-Pascual et al. 2021), and complex differences exist between developed and developing countries (Nuñez and Pauchard 2010). However, quantitative studies have mostly focused on a subset of these factors and on specific parts of the world. For example, national wealth, human population and the Human Development Index (HDI) [United Nations Development Programme (UNDP)] have been shown to be positively correlated with alien species richness in Europe and North Africa (Vilà and Pujadas 2001; Pyšek et al. 2010), while at the same time countries with low HDI have a severely limited capacity to manage biological invasions and mitigate their impacts (Early et al. 2016). In addition, it has been shown that the effect of Governance (the capacity of the government to effectively formulate and implement sound policies; Table 1) is diminished in countries with high per capita GDP in Eurasia (Evans et al. 2018). Low levels of governance and high levels of corruption have been associated with higher exports of alien species, as outbound pathways are poorly regulated and subsequently lead to greater potential rates of introduction in importing countries (Brenton-Rule et al. 2016). Quantitative analyses are nonetheless scarce for political, legal, social and technological predictors of biological invasions, especially at the global scale, as many analyses have focused on developed countries only (Nuñez and Pauchard 2010).

**Table 1:** Indices characterising the main factors essential to explain biological invasions (as identified by Roura-Pascual et al. 2021) and their corresponding component variables

Index	Relationship with biological invasions	Variable	Description	Source
Governance	The political context influences the capacity of a country to vote and apply appropriate policies and management actions to control and prevent the introduction of Invasive Alien Species (IAS)	Rule of Law	Perception of the extent to which the population has confidence in and abides by the rules of society, and in particular the quality of contract enforcement, property rights, the police, and the courts, as well as the likelihood of crime and violence	Kaufmann et al. (2010) and The World Bank (2019)
		Government Effectiveness	Perception of the quality of public services, the quality of the civil service and the degree of its independence from political pressures, the quality of policy formulation and implementation, and the credibility of the government's commitment to such policies	Kaufmann et al. (2010) and The World Bank (2019)
		Voice and Accountability	Perception of the extent to which a country's citizens are able to participate in selecting their government, as well as freedom of expression, freedom of association and a free media	Kaufmann et al. (2010) and The World Bank (2019)
		Control of Corruption	Perception of the extent to which public power is exercised for private gain, including both petty and grand forms of corruption, as well as "capture" of the state by elites and private interests	Kaufmann et al. (2010) and The World Bank (2019)
		Political Globalization*	Summary of the diffusion of countries’ government policies and the ability to engage in international political cooperation	Dreher (2006) and Gygli et al. (2019)
Trade	The importation of goods and services into a country facilitates the introduction of propagules	Imports in Good and Services	Value of all goods and other market services received from the rest of the world	The World Bank (2019)
		Per capita Gross National Income (GNI)*	Sum of a country’s gross domestic product (GDP) plus net income (positive or negative) from abroad, divided by population size	The World Bank (2019)
Environmental Performance	Environmental conditions, including disturbance, land cover, pollution, etc., can influence the establishment of alien species	Environmental Performance Index (EPI)	Summary of the state of sustainability of countries, based on 32 performance indicators across 11 issue categories (Biodiversity and habitat, Ecosystem services, Fisheries, Water resources, Climate change, Pollution emissions, Agriculture, Waste management, Heavy metals, Sanitation and drinking water and Air quality)	Hsu et al. (2014) and Yale Center for Environmental Law and Policy-YCELP-Yale University et al. (2014)
Lifestyle and Education	People’s lifestyle, their connections with other cultures and therefore geographical areas, itself influenced by education, can move and introduce propagules to novel environments. Lifestyle and education are also likely linked to other predictors, such as governance	Average level of Education of the Population	Average of the maximum level of education across countries’ inhabitants, using a scale from 1 to 5 to quantify levels of education	European Commission and Joint Research Centre (2018)
		Information Globalization Index (de jure)	Summary of countries’ ability to share information with other countries	Dreher (2006) and Gygli et al. (2019)
		Cultural Globalization Index (de jure)	Summary of countries’ openness towards and the ability to understand and adopt foreign cultural influences	Dreher (2006) and Gygli et al. (2019)
Innovation	Technological innovations can offer means to control and prevent the introduction of alien species, but may also facilitate trade activities and contribute to impact the environment	Global Innovation Index	Summary of countries’ capacity for, and success in, innovation, based on variables from multiple sources	Cornell University et al. (2019)

Note that variables with * were discarded from analyses (see “Methods”)

Understanding how country-level socio-economic and environmental factors together shape the current and future state of biological invasions at the global scale is crucial to capture future dynamics of biological invasions in global scenarios (Lenzner et al. 2019; Roura-Pascual et al. 2021). For example, trade promotes species introduction (Seebens et al. 2015; Hulme 2021), and environmental conditions affect the capacity of these introduced species to establish (Hobbs and Huenneke 1992). Technological advancements can offer the means to better intercept species before they are introduced, or to better eradicate established species (Begley et al. 2020; Martinez et al. 2020). General factors such as governance levels also affect the design and implementation of efficient specific policies and management actions, and therefore the capacities to influence EAS richness (Evans et al. 2018). Recent global studies considering the combined role of social, political, environmental and economic predictors for the future of biological invasions have mostly relied on expert knowledge (Essl et al. 2020b; Lenzner et al. 2020). In addition, quantitative and modelling approaches are often species-specific, and tend to focus on environmental factors such as climate change or disturbance (e.g. Bradley et al. 2010; Bertelsmeier et al. 2016). Therefore, there is a need for a comprehensive quantitative assessment of these relationships.

Here, we compare 125 countries across all continents (see map in Figure S1) against a set of socio-economic and environmental indices. These indices represent factors that are considered essential to understand and project the future of biological invasions at a global scale, because they underpin different, context-dependent mechanisms of invasion and management possibilities, while being general enough to allow for a global comparison between countries (Essl et al. 2020b; Roura-Pascual et al. 2021). For each country, we quantify recent and—if available—historical conditions using five indices (Governance, Trade, Environmental Performance, Lifestyle and Education, and Innovation; Table 1). We (1) examine the relationships between these indices and then relate their (2) recent (2015) and (3) past (1996 or averaged over 1996–2015—for the predictors with available historical records, i.e. Governance and Trade) values to EAS richness per country. As a response variable, we use country-level EAS richness of eight taxonomic groups (plants, ants, amphibians, reptiles, fishes, birds, mammals and spiders) based on the most comprehensive country-level data set on EAS richness (Dawson et al. 2017a). Moreover, we relate these indices to the national response capacities to manage and mitigate biological invasions and their impacts presented in Early et al. (2016).

Based on the results from these analyses, we show how Governance and Trade can be used to identify a two-dimensional socio-economic space describing the capacity (or lack thereof) of countries to mitigate the spread, establishment and impact of alien species. We assess how different geographic regions (Figure S1) perform in this socio-economic space. Finally, we explore how countries and geographic regions have changed their position in this socio-economic space since 1996, and explain why capturing divergences between country trajectories through time is crucial to understanding the dynamics of biological invasions and to capture the main challenges countries are currently facing to reduce invasions and limit their impacts in the future.

## Material and methods

### Predictor selection and data

Based on previous findings (Essl et al. 2020b; Roura-Pascual et al. 2021), we selected five groups of socio-economic and environmental variables characterising factors considered to be essential to understand and project future invasion dynamics (Table 1):

1. Governance, i.e. the capacity of a country to design and implement policies, including policies aimed at addressing biological invasions;
2. Trade, as the most important predictor of propagule pressure;
3. Environmental Performance, i.e. a measure of environmental health and ecosystem vitality, including land use, which influences the capacity of alien species to successfully establish and spread in a novel environment;
4. Lifestyle and Education, i.e. factors influencing people’s values and perception of nature, their understanding of environmental issues (including biological invasions) and their connections with other cultures and countries, with implications for alien species dispersal and establishment, e.g. via recreational activities and tourism, or mode of consumption;
5. Innovation, i.e. technological progress which can enhance the knowledge and technological means to detect, prevent and manage biological invasions.

To quantify each factor, we searched for data available at the country scale from open access repositories with good transparency about the methods used to collate these variables, to ensure data quality and long-term maintenance. This resulted in a total of 12 variables extracted from the World Bank data repository (The World Bank 2019), the KOF Swiss Economic Institute (Dreher 2006; Gygli et al. 2019), the Global Innovation Index (Cornell University et al. 2019) and the Wittgenstein Centre for Demography and Global Human Capital (European Commission and Joint Research Centre 2018) (Table 1).

We extracted data on the selected variables for 2015 (referred to as ‘recent data’ hereafter), as this year corresponded to the final year for which data of the response variable, EAS richness, have been considered in our data set (see below). When data for this year were not available for a country, we used data from the most recent preceding year until 2010. To explore potential legacies of historical predictor conditions, we extracted historical data for Governance and Trade for each year from 1996 onwards, which was the first year for which these data were available for Governance; for the other groups of variables, data were available only for the more recent years. Altogether, data were available for 125 countries, which were then considered in the analyses (excluding overseas territories and territories separate from mainland, such as the French Caribbean or Hawaii, which often have different invasion dynamics; see Figure S1).

Further, following Dawson et al. (2017a, b) we extracted mean annual temperature (BIO1) and mean annual precipitation (BIO12) for the years 1960 to 1990 from WorldClim (http://www.worldclim.org); for each country, we calculated mean annual temperature and total annual precipitation as the mean of raster cells within country borders. To control for the effect of area on species richness, we included country area (The World Bank 2019) as an additional predictor variable in our models. As different countries may have better data on alien species than others, sampling effort was also included. Sampling effort was measured using the metric proposed by Meyer et al. (2015), which is based on the number of records per unit area mobilised from the Global Biodiversity Information Facility (GBIF) and accounting for native species richness (Dawson et al. 2017b). For reptiles, fishes and spiders, taxon-specific sampling effort was not available. In the following, we refer to these variables as “non-anthropogenic variables”, to distinguish them from the indices characterising the five factors described above.

### Established alien species richness data

We calculated country-specific levels of invasion based on data of EAS richness of eight taxonomic groups for which global distribution data were available (plants, ants, amphibians, reptiles, fishes, birds, mammals and spiders) (Dawson et al. 2017a). Following Dawson et al. (2017a), overall EAS richness was calculated by converting absolute EAS richness to a relative scale by dividing species richness by the maximum richness over all countries, resulting in values ranging from 0 to 1. Overall EAS richness for each country was then computed as the mean of relative richness values across taxonomic groups. Although data for predictor variables were only selected for 1996 onward, we used cumulative alien species numbers rather than data on species introduced after 1996 only because we assume there is a continuity in explanatory variables over time, and most alien species were introduced after 1950 (Seebens et al. 2017). In addition, by using cumulative numbers the influence of a reporting lag, which led to lower records in more recent years (Seebens et al. 2017), is minimized.

### National response capacity data

Data representing countries’ capacity for reactive and proactive responses to invasive alien species (IAS, the subset of EAS with negative environmental, social or economic impacts) were obtained from Early et al. (2016). National proactive capacity assesses the capacity of a country to prevent or contain early the emerging incursions by IAS. National reactive capacity accounts for the expertise, resources and willingness to mitigate the damage from IAS that is present in a country, which is essential to make IAS policy effective.

### Variable selection

The 12 socio-economic and environmental variables selected to describe the main factors considered essential to explain EAS richness per country (i.e. excluding the other, non-anthropogenic variables; Table 1) were interrelated in complex ways, resulting in collinearities. To keep groups of variables as independent from each other as possible and better disentangle their respective effects on the response variables described below, we imposed internal coherence between variables used to characterize a given factor. In other words, to be coherent, variables characterizing a factor had to be more correlated with each other than with variables characterizing other factors (e.g. a variable characterising Governance had to be more correlated with the other variables characterising Governance than with variables characterising Trade, Environmental Performance, Lifestyle and Education, or Innovation). Variables that characterize a given factor but are more correlated with variables characterising another factor likely indicate causal relationships between specific aspects of the two factors that would cause a high correlation between indices if they were included as predictors of EAS richness. Although understanding the causal relationships between these factors and the effects on biological invasions is interesting, this is beyond the scope of this study. Rather, maximizing independence between factors allows us to better disentangle their respective effects on the response variables described below, whereas high correlations would lead to similar results in the analyses, rendering the analysis of the relationship difficult to interpret. We therefore discarded political globalization (initially considered to characterize Governance), which was more strongly correlated with imports of goods and services (characterizing Trade) than with any of the other variables characterising Governance. We also discarded per capita Gross National Income (initially considered to characterize Trade), which was more strongly correlated with control of corruption, government effectiveness and rule of law (characterizing Governance) than with imports of goods and services. The remaining 10 socio-economic and environmental variables were standardized to zero mean and unit standard deviation. Variables characterizing each factor were then averaged to generate indices that were used as predictors of EAS richness for each country and year: Governance was quantified as the mean of the Rule of Law, Government Effectiveness, Voice and Accountability, and Control of Corruption indicators; Trade was measured as total imports in Good and Services; Environmental Performance was measured by the Environmental Performance Index, which includes land use; Lifestyle and Education was quantified as the mean of the average level of Education of a Population, the Information Globalization Index and the Cultural Globalization Index; Innovation was measured by the Global Innovation Index (Table 1). By using this variable selection protocol, we avoided potential collinearity issues, reduced complexity and facilitated the interpretation of results (Dormann et al. 2013).

### Analyzing the relationships between indices and established alien species richness

We investigated the relationship between the five indices and EAS richness per country with linear mixed-effects models (LMMs) using the lmer function from the lme4 R package v.1.1-27 (Bates et al. 2015; R Core Team 2019). To statistically identify non-linearities observed in preliminary analyses using splines, we fitted linear, second-order (quadratic) and third-order (cubic) models for each individual index. Quadratic models enabled us to detect accelerating (i.e. positive coefficients) or decelerating (i.e. negative coefficients) relationships. Similarly, we used cubic models to identify both accelerating and decelerating relationships across the range of values for an index (for example, steepest slopes could be expected at intermediate values, and saturation could be expected at high or low values). We used polynomials rather than more complex regression models, such as generalised additive models, to better assess the significance of non-linearities in the relationships.

We also incorporated the non-anthropogenic variables described above (i.e. mean annual temperature, total annual precipitation, mainland or island status of the country [represented by a categorical variable], country area, sampling effort [ln-transformed] and its interaction with country area [or only country area when sampling effort was not available for a taxonomic group]) as fixed effects. We used overall EAS richness (ln-transformed to satisfy assumptions of normality of residuals and variance homogeneity) and EAS richness of each taxonomic group individually (ln [EAS richness + 1] transformed) as response variables. To account for spatial autocorrelation, we used broad geographical regions (level 1 of the Biodiversity Information Standards – TDWG, (45)) as random effects. Alternative generalized linear mixed models using binomial and Poisson link functions on untransformed response variables provided qualitatively similar results (not shown), but could not be tested for spatial autocorrelation due to long computation times.

For each index X, we therefore assessed the following three models:

1 $$\mathrm{Linear}{:}\, S \sim X+A+E+A\times E+T+P+M+\left(1|TDWG1\right),$$

2 $$\begin{aligned} &{\text{Quadratic:}}\; S \sim {X}^{2}+X+A+E+A \\& \quad \times E+T+P+M+\left(1|TDWG1\right), \end{aligned}$$

3 $$\begin{aligned} \mathrm{Cubic}{:}\,& S \sim {X}^{3}+{X}^{2}+X+A+E+A\\& \times E+T+P+M+\left(1|TDWG1\right), \end{aligned}$$

where *S* is EAS richness, *X* is a socio-economic or environmental index, *A* is country area, *E* is sampling effort (*A* was used instead of *A* + *E* + *A* × *E* for reptiles, fishes and spiders, for which sampling effort was not available), *T* is mean annual temperature, *P* is mean annual precipitation, *M* is mainland or island status and TDWG1 is the level 1 of the Biodiversity Information Standards. To avoid issues of data dredging, and because the focus of this study is on socio-economic and environmental factors, we only considered linear relationships for the non-anthropogenic variables.

We assessed model performance using the Akaike Information Criterion corrected for small-sample size (AICc) (Cavanaugh 1997), computed with the AICc function in the AICcmodavg R package v2.3-1 (Mazerolle 2020), and using the marginal variance explained after accounting for random effects, computed with the r2beta function in the r2glmm R package v0.1.2 (Jaeger 2017). We used the AICc because we are comparing nested and non-nested models for the same response variables and number of data points. The AICc therefore allows to compare models while accounting for the additional complexity generated by the inclusion of the indices quantifying Governance, Trade, Environmental Performance, Lifestyle and Education, or Innovation in addition to the non-anthropogenic variables, and by the use of higher order polynomials. We compared all models for each response variable (i.e. for each taxonomic group richness and the overall richness metric). Importantly, we were interested in the ΔAICc values (1) between the linear, quadratic and cubic equations for the same index used as a predictor, and (2) between the models with the lowest AICc value for the different indices and for the model including no index as a predictor (Table 2). Due to the large number and variety of models compared, we do not report all ΔAICc values, as this would be intractable, but we indicate the relevant ΔAICc values in tables, to assess model performance. A model was considered more performant for ΔAICc > 4, although this threshold was used for convenience, and results should be interpreted in a more continuous fashion with models having more or less support (Burnham et al. 2011).

**Table 2:** Results of model fitting for explaining EAS richness and national capacities in 125 countries based on the small-sample size corrected Akaike Information Criterion (AICc) for 2015

Response variable	Governance	Trade	Environmental performance	Lifestyle and education	Innovation
All taxa combined *r*^2^ = 0.17 ΔAICc = 53.06	*Cubic* *r*^*2*^ = *0.24* *ΔAICc*_*all*_ = *52.84* *ΔAICc*_*lin*_ = *0.65*	**Quadratic** **r**^**2**^** = 0.37** **ΔAICc**_**all**_** = 0** **ΔAICc**_**lin**_** = 23.4**	*Linear* *r*^*2*^ = *0.26* *ΔAICc*_*all*_ = *45.71* *ΔAICc*_*lin*_ = *0*	Linear *r*^2^ = 0.25 ΔAICc_all_ = 50.24 ΔAICc_lin_ = 0	*Quadratic* *r*^*2*^ = *0.23* *ΔAICc*_*all*_ = *51.07* *ΔAICc*_*lin*_ = *3.4*
Plants *r*^2^ = 0.42 ΔAICc = 10.09	Cubic *r*^2^ = 0.45 ΔAICc_all_ = 4.34 ΔAICc_lin_ = 8.56	**Quadratic** **r**^**2**^** = 0.48** **ΔAICc**_**all**_** = 0** **ΔAICc**_**lin**_** = 4.54**	Cubic *r*^2^ = 0.44 ΔAICc_all_ = 5.29 ΔAICc_lin_ = 7.36	Quadratic *r*^2^ = 0.46 ΔAICc_all_ = 4.09 ΔAICc_lin_ = 4.03	Quadratic *r*^2^ = 0.42 ΔAICc_all_ = 9.03 ΔAICc_lin_ = 5.58
Ants *r*^2^ = 0.34 ΔAICc = 12.16	Cubic *r*^2^ = 0.36 ΔAICc_all_ = 7.65 ΔAICc_lin_ = 8.73	Quadratic *r*^2^ = 0.38 ΔAICc_all_ = 3.4 ΔAICc_lin_ = 4.3	Quadratic *r*^2^ = 0.4 ΔAICc_all_ = 4.72 ΔAICc_lin_ = 6.73	**Quadratic** **r**^**2**^** = 0.36** **ΔAICc**_**all**_** = 0** **ΔAICc**_**lin**_** = 16.44**	Cubic *r*^2^ = 0.36 ΔAICc_all_ = 8.25 ΔAICc_lin_ = 5.29
Amphibians *r*^2^ = 0.44 ΔAICc = 14.33	Cubic *r*^2^ = 0.49 ΔAICc_all_ = 8.94 ΔAICc_lin_ = 8.28	**Quadratic** **r**^**2**^** = 0.53** **ΔAICc**_**all**_** = 0** **ΔAICc**_**lin**_** = 7.64**	Cubic *r*^2^ = 0.49 ΔAICc_all_ = 8.42 ΔAICc_lin_ = 4.01	Cubic *r*^2^ = 0.47 ΔAICc_all_ = 11.7 ΔAICc_lin_ = 5.97	*Quadratic* *r*^*2*^ = *0.46* *ΔAICc*_*all*_ = *12.53* *ΔAICc*_*lin*_ = *3.27*
Reptiles *r*^2^ = 0.17 ΔAICc = 32.72	Cubic *r*^2^ = 0.23 ΔAICc_all_ = 21.25 ΔAICc_lin_ = 10.32	**Cubic** **r**^**2**^** = 0.38** **ΔAICc**_**all**_** = 0** **ΔAICc**_**lin**_** = 20.78**	*Quadratic* *r*^*2*^ = *0.18* *ΔAICc*_*all*_ = *23.82* *ΔAICc*_*lin*_ = *3.89*	Quadratic *r*^2^ = 0.16 ΔAICc_all_ = 31.05 ΔAICc_lin_ = 4.11	Quadratic *r*^2^ = 0.2 ΔAICc_all_ = 23.24 ΔAICc_lin_ = 10.28
Fishes *r*^2^ = 0.21 ΔAICc = 48.38	Cubic *r*^2^ = 0.32 ΔAICc_all_ = 34.21 ΔAICc_lin_ = 6.38	**Quadratic** **r**^**2**^** = 0.52** **ΔAICc**_**all**_** = 0** **ΔAICc**_**lin**_** = 4.04**	Cubic *r*^2^ = 0.4 ΔAICc_all_ = 20.96 ΔAICc_lin_ = 4.28	Quadratic *r*^2^ = 0.31 ΔAICc_all_ = 34.97 ΔAICc_lin_ = 6.1	Quadratic *r*^2^ = 0.34 ΔAICc_all_ = 33.58 ΔAICc_lin_ = 5.89
Birds *r*^2^ = 0.36 ΔAICc = 36.45	Quadratic *r*^2^ = 0.43 ΔAICc_all_ = 25.2 ΔAICc_lin_ = 4.79	**Quadratic** **r**^**2**^** = 0.47** **ΔAICc**_**all**_** = 0** **ΔAICc**_**lin**_** = 4.86**	*Quadratic* *r*^*2*^ = *0.51* *ΔAICc*_*all*_ = *15.95* *ΔAICc*_*lin*_ = *3.65*	Quadratic *r*^2^ = 0.52 ΔAICc_all_ = 20.19 ΔAICc_lin_ = 6.13	Cubic *r*^2^ = 0.43 ΔAICc_all_ = 28.81 ΔAICc_lin_ = 7.57
Mammals *r*^2^ = 0.36 ΔAICc = 17.91	*Cubic* *r*^*2*^ = *0.48* *ΔAICc*_*all*_ = *8.18* *ΔAICc*_*lin*_ = *3.79*	Quadratic *r*^2^ = 0.41 ΔAICc_all_ = 8.74 ΔAICc_lin_ = 5.21	*Quadratic* *r*^*2*^ = *0.52* *ΔAICc*_*all*_ = *2.72* *ΔAICc*_*lin*_ = *3.72*	***Quadratic*** ***r***^***2***^** = *0.5*** ***ΔAICc***_***all***_** = *0*** ***ΔAICc***_***lin***_** = *2.69***	*Quadratic* *r*^*2*^ = *0.39* *ΔAICc*_*all*_ = *17.62* *ΔAICc*_*lin*_ = *3.5*
Spiders *r*^2^ = 0.14 ΔAICc = 30.39	Cubic *r*^2^ = 0.25 ΔAICc_all_ = 16.06 ΔAICc_lin_ = 8.29	**Quadratic** **r**^**2**^** = 0.32** **ΔAICc**_**all**_** = 0** **ΔAICc**_**lin**_** = 7.49**	*Quadratic* *r*^*2*^ = *0.28* *ΔAICc*_*all*_ = *14.77* *ΔAICc*_*lin*_ = *3.97*	Quadratic *r*^2^ = 0.32 ΔAICc_all_ = 12.69 ΔAICc_lin_ = 5.34	Cubic *r*^2^ = 0.19 ΔAICc_all_ = 23.86 ΔAICc_lin_ = 4.71
National proactive capacity ΔAICc = 23.69	***Quadratic*** ***r***^***2***^** = *0.24*** ***ΔAICc***_***all***_** = *0*** ***ΔAICc***_***lin***_** = *2.6***	Quadratic *r*^2^ = 0.09 ΔAICc_all_ = 13.26 ΔAICc_lin_ = 5.05	Quadratic *r*^2^ = 0.17 ΔAICc_all_ = 11.09 ΔAICc_lin_ = 5.21	*Quadratic* *r*^*2*^ = *0.3* *ΔAICc*_*all*_ = *3.74* *ΔAICc*_*lin*_ = *2.52*	Quadratic *r*^2^ = 0.13 ΔAICc_all_ = 10.2 ΔAICc_lin_ = 9.14
National reactive capacity ΔAICc = 22.4	*Quadratic* *r*^*2*^ = *0.11* *ΔAICc*_*all*_ = *17.07* *ΔAICc*_*lin*_ = *1.98*	*Quadratic* *r*^*2*^ = *0.2* *ΔAICc*_*all*_ = *3.04* *ΔAICc*_*lin*_ = *1.89*	*Quadratic* *r*^*2*^ = *0.15* *ΔAICc*_*all*_ = *14.8* *ΔAICc*_*lin*_ = *1.91*	***Quadratic*** ***r***^***2***^** = *0.31*** ***ΔAICc***_***all***_** = *0*** ***ΔAICc***_***lin***_** = *1.65***	*Quadratic* *r*^*2*^ = *0.13* *ΔAICc*_*all*_ = *14.47* *ΔAICc*_*lin*_ = *3.43*

In normal bold are the models with the lowest ΔAICc values over all combinations of predictors. ΔAICc_all_ is the difference with the lowest AICc values over all predictors and all polynomial forms. ΔAICc_lin_ is the difference with the AICc value of the linear model for the same predictor. In italic are the polynomial models for which the lowest ΔAICc is not larger than 4 compared to the linear model including the same predictor, and for which the linear model can therefore be considered as performing better. *r*^2^ values are the marginal variance. Values in the first column are those when only non-anthropogenic predictors and random effects were included (the marginal *r*^2^ is therefore 0 for the national capacity models, as only random effects are included)

We also computed LMMs incorporating combinations of indices as predictors in the models using the linear, quadratic and cubic transformations (Eqs. 4–6 show combinations of Governance—G—and Trade—Tr). Governance, Environmental Performance and Lifestyle and Education were highly positively correlated (0.67 ≤ *r* ≤ 0.80) across countries, but less so with Trade (*r* ≤ 0.6). Innovation was moderately correlated with all other indices (0.61 ≤ *r* ≤ 0.64). A principal component analysis (PCA) confirmed the distinction between these three groups of indices (first group: Governance, Environmental Performance, Lifestyle and Education; second group: Trade; third group: Innovation; Figure S2g). Based on these correlation values, in our models we only combined Trade with either Governance, Environmental Performance or Lifestyle and Education as predictors, to avoid collinearity issues. Incorporating more than two predictors in exploratory analyses led to variance inflation factors > 3 in the models (results not shown). We did not use the principal components of the PCA as predictors for two reasons: (1) To do so would have prevented the exploration of the effects of historical data due to lack of data for other predictors; (2) we were better able to explore the effects of the different indices on the different response variables we considered (see national response capacities below).

4 $$\begin{aligned} \mathrm{Linear}{:}\, &S \sim G+Tr+A+E+A \\& \times E+T+P+M+\left(1|TDWG1\right), \end{aligned}$$

5 $$\begin{aligned} \mathrm{Quadratic}{:}\, &S \sim {G}^{2}+G+{Tr}^{2}+Tr+A+E+A\\& \times E+T+P+M+\left(1|TDWG1\right), \end{aligned}$$

6 $$\begin{aligned} \mathrm{Cubic}{:}\,& S \sim {G}^{3}+{G}^{2}+\mathrm{G}+{ Tr}^{3}+{Tr}^{2}+Tr+A\\& +E+A \times E+T+P+M+\left(1|TDWG1\right).\end{aligned}$$

Finally, for models using Governance and Trade as predictors, we performed analyses for historical conditions for 1996 and for the annual values averaged between 1996 and 2015 (historical data were not available for the other three indices). As we describe further, Governance and Trade have changed quite substantially over the 20 years for which the data was available for different countries, often in different directions (e.g. Governance could increase or decrease depending on the country). Since the time frame of the historical legacy of these factors on biological invasions is uncertain, we used the 1996–2015 average to capture a longer time period than a single year, but also 1996 only as it may be more representative of older lag times.

The same 125 countries were used in all analyses, permitting comparison with respective models using the 2015 data. A lower AICc value for models using historical data than using 2015 data (here using ΔAICc > 4 as a threshold) would reveal the historical legacy of these predictors for EAS richness.

### Analyzing the relationships between indices and national response capacities

We examined how the five indices characterizing essential factors for explaining invasions also explained the ability of countries to control and manage biological invasions, i.e. their national response capacities (Early et al. 2016). This is important to project future levels of biological invasions and to disentangle how these indices influence the management of EAS from their introduction, establishment and spread. We modified Eqs. 1–6 using proactive and national reactive capacities as response variables and removed the non-anthropogenic variables (T, P, M, A, E) as predictors of EAS richness (Eqs. 7–12). All indices may be related to national response capacities due to complex feedbacks between variables. For example, biosecurity measures can influence how trade is conducted, and environmental conditions can increase invasion risks at the different stages of invasion, in turn leading to the adoption of control and management measures. We hypothesized that Governance, Environmental Performance and Lifestyle and Education should especially be positively correlated with the proactive capacity of a country to prevent or rapidly respond to emerging incursions by IAS. Governance and Lifestyle and Education should reflect the proactive mindset of people and governments to address environmental issues, measured by Environmental Performance. In contrast, as Trade is expected to lead to more species introductions (Seebens et al. 2015), which in turn should lead to more reactive measures due to rising awareness of the impacts of IAS, we argue that Trade should show a stronger correlation with the reactive capacity of countries to mitigate negative impacts caused by IAS already present. As for EAS richness, models were evaluated with recent (2015) and historical (1996) predictor data, and averaged over the 1996–2015 period.

7 $$\mathrm{Linear}{:}\, C \sim X+\left(1|TDWG1\right),$$

8 $$\mathrm{Quadratic}{:}\, C \sim {X}^{2}+X+\left(1|TDWG1\right),$$

9 $$\mathrm{Cubic}{:}\, C \sim {X}^{3}+{X}^{2}+X+\left(1|TDWG1\right),$$

10 $$\mathrm{Linear}{:}\, C \sim G+Tr+\left(1|TDWG1\right),$$

11 $$\mathrm{Quadratic}{:}\, C \sim {G}^{2}+G+{Tr}^{2}+Tr+\left(1|TDWG1\right),$$

12 $$\mathrm{Cubic}{:}\, C \sim {G}^{3}+{G}^{2}+G +{Tr}^{3}+{Tr}^{2}+Tr+\left(1|TDWG1\right),$$

where *C* is national proactive or reactive capacity and the other notations are as in Eqs. 7–12. As for EAS richness, we also combined Trade with Environmental Performance and with Lifestyle and Education for 2015.

For all models, we tested for residual spatial autocorrelation by constructing correlograms of Moran’s I in relation to increasing distance between country centroids using the spline.correlog function in the ncf R package v1.2-9 (Bjornstad 2020). Significance was assessed using 95% confidence intervals, built from 1000 bootstrapped randomizations of the residuals (Figures S7, S8). All statistical analyses were performed with the R software v. 4.0.3 (R Core Team 2019).

### Visualization of countries in a two-dimensional socio-economic space

We mapped the trajectories of countries through time in a two-dimensional socio-economic space defined by Governance and Trade through time by taking into account the full range of data available for Governance and Trade (i.e. from 1996 to 2018). Although we are not directly assessing how changes in the position of a country through time is linked to changes in EAS richness, the results from the regression analyses for all countries can be interpreted in a space-for-time substitution fashion, and allow us to discuss how future levels of biological invasions may be reflected by changes of position in this socio-economic space. Making future projections is beyond the scope of this study, but observing how country positions have changed through time over the past 20 years can offer insights and avenues for further discussions about future biological invasions.

To facilitate the interpretation of results, countries were assigned to different geographic regions. To identify groups of countries that differ distinctly from each other in 2015 (the most recent year for which EAS richness and national response capacity data were both available), we applied two hierarchical clustering algorithms based on distance between countries in this socio-economic space. We used the complete-linkage and the Ward methods in the R function hclust from the default stats package. To evaluate the number of clusters best separating the countries, we used the function NbClust from the NbClust R Package v3.0 (Charrad et al. 2014), which evaluates the number of clusters based on 30 different indices. Visualizations of country trajectories through time were created with Gapminder (free material from http://www.gapminder.org).

## Results

### Predictors and numbers of established alien species in countries

Using recent (2015) data on predictors, model comparison revealed that all models including one of the five indices as a predictor better explained observed overall richness of EAS than models with only non-anthropogenic variables included and none of the five indices (ΔAICc > 10), increasing marginal *r*^2^ values by up to 31% in absolute values (Table 2). Trade was the best predictor of richness for overall EAS data and for most individual taxonomic groups, i.e. plants, amphibians, reptiles, fishes, birds and spiders (Table 2, Figure S3). Meanwhile, for ants and mammals, Lifestyle and Education was the most important predictor. The improvement in model performance gained from including socio-economic or environmental indices as predictors varied between taxonomic groups, being negligible for ants and doubling the marginal variance for fishes. The relationships between socio-economic and environmental indices and EAS richness were non-linear for most taxa and indices (ΔAICc > 4; Table 2), mammals being the largest exception. For Trade, the relationship indicated either a constant increase or an acceleration of EAS richness as Trade increased for all taxonomic groups (Figure S3). For Innovation, the relationship was also accelerating for most taxa. In contrast, the relationships between EAS richness and Governance and Lifestyle and Education were either quadratic or cubic and tended to decrease or decelerate at high values (Table 2, Figure S3). For Environmental Performance, the relationship was more variable across taxonomic groups, with half of them showing a decrease in EAS at high values (Figure S3).

**Fig. 1:**
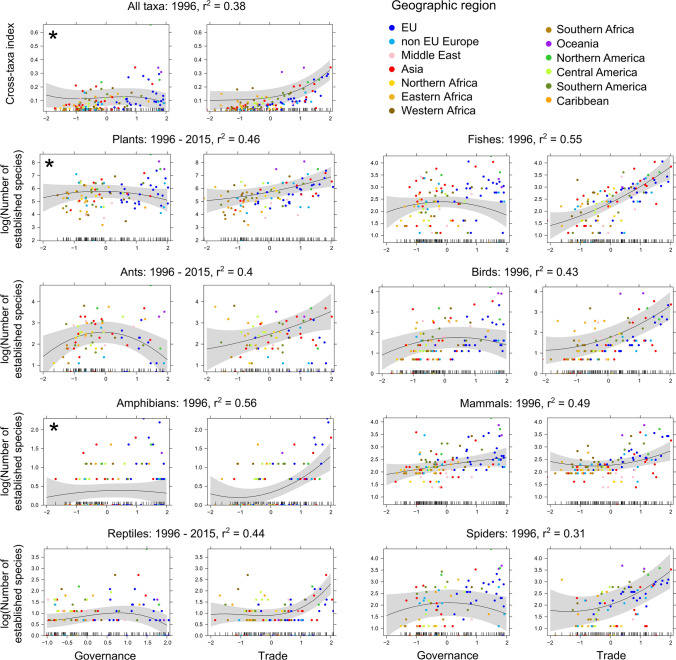
Relationships between the Governance and Trade indices and the number of established alien species (EAS) in 125 countries, when both indices were included as predictors in linear mixed-effects models. For each taxonomic group, the period generating the lowest AICc was selected (i.e. using data for 1996 or averaged over the 1996–2015 period; see Tables S1–S3 for AICc value comparisons), and the marginal *r*^2^ is reported. Different colors indicate geographic regions the countries belong to. Asterisks indicate that a predictor did not improve a model (i.e. a one-predictor model had a lower AICc than a two-predictor model). Rug plots on the inside of the *X*‐axes show the distributions of the data points along individual predictor gradients

Two-way combinations of 2015 Trade with Governance, Lifestyle and Education, or Environmental Performance (i.e. additive models containing two least-correlated predictors) tended to explained EAS richness better than individual predictors (lower AICc values, although the ΔAICc values between the best two-predictor and one-predictor models [in addition to the non-anthropogenic variables] were > 4 for ants, birds, mammals and spiders only; Table S1). Combinations of Trade and Governance for historical data (i.e. 1996 or averaged over 1996–2015; past data was not available for the other indices), explained EAS richness better than their combinations in 2015 (lower AICc values, although the ΔAICc values between the past and 2015 models was < 4 for plants and ants; Fig. 1; Table S2). Models only including historical Trade resulted in the best-fitting models for plants and amphibians, and for overall EAS richness (lower AICc values). For the other models, ΔAICc values between two-predictor and one-predictor models was > 4 for ants, birds and spiders only (see also variation partitioning analyses; Figure S5). When comparing combinations of Trade with Governance, Lifestyle and Education or Environmental Performance in 2015 and combinations of Trade with Governance for historical data, the combination of Trade with Lifestyle and Education for 2015 generated the best fitting models for ants, mammals and spiders (Tables S1, S3).

### Predictors and national response capacities

For national proactive capacity to prevent or rapidly respond to emerging IAS, using Governance as a predictor generated the lowest AICc value, but Lifestyle and Education generated the highest marginal *r*^2^ compared to other indices for the 2015 data (Table 2, Figure S4). For national reactive capacity, i.e. the expertise, resources and willingness to mitigate negative impacts caused by IAS, Lifestyle and Education was the best predictor, with the lowest AICc and highest marginal *r*^2^ (Table 2). Although quadratic models including positive terms (i.e. indicating a disproportionately strong increase in national capacity with increasing index values) consistently generated lower AICc values than linear ones for all indices (Table 2), the ΔAICc values were mostly < 4, and linear relationships can be considered as more parsimonious (Figure S4).

**Fig. 2:**
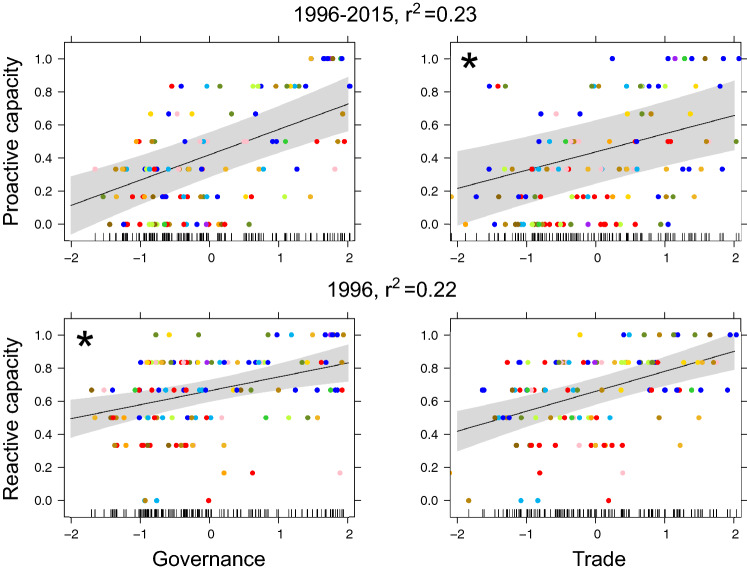
Relationships between Governance and Trade, and national capacities to mitigate the impacts of biological invasions, when both indices were included as predictors in linear mixed-effects models. The year or combination of years generating the lowest AICc were selected (see Tables S1–S3 for AICc value comparisons), and the marginal *r*^2^ is reported. Different colors indicate the geographic regions countries belong to. Asterisks indicate that the predictor did not significantly explain established alien species richness (i.e. when linear models with a single predictor generated a lower AICc than when both predictors were included). Rug plots on the inside of the *X*‐axes show the distributions of the data points along individual predictor gradients

Average Governance between 1996 and 2015 explained more variance than any other model incorporating Governance or Trade for proactive capacity (Table S3). This model showed an increase of national proactive capacity with better Governance (Fig. 2). When considering only Governance and Trade (to compare recent and historical data), Trade for 1996 was the best predictor for reactive capacity (Table S2), but Lifestyle and Education in 2015 had a lower AICc and higher r^2^.

### Mapping countries according to national levels of predictors of invasions

**Fig. 3:**
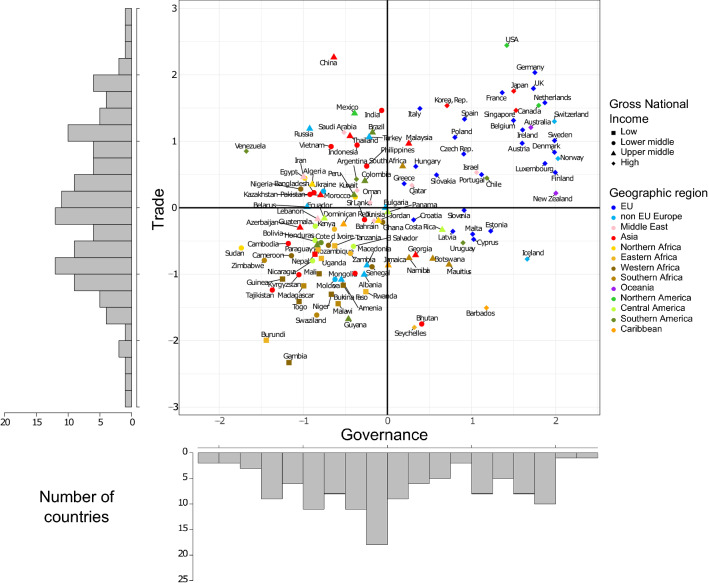
The 125 countries organized in the two-dimensional socio-economic space based on recent (2015) Governance and Trade data. The histograms show the distribution of countries based on Governance and Trade. The bold horizontal and vertical lines indicate the origin axes, which correspond to the centroid of the country distribution. Gross National Income categories are based on the World Bank classification (The World Bank 2019)

The five indices considered here were interrelated, but Governance and Trade were the least correlated indices (*r*^2^ = 0.49; Figure S2). Since their historical values were also always better predictors of EAS richness and national capacities than their recent values, and often amongst the best predictors in general, we selected Governance and Trade to map countries in a two-dimensional space defined by these two indices (Fig. 3). This two-dimensional approach hence represents the realized socio-economic space of country positions with respect to the factors that have the highest impacts on biological invasion, either by limiting their introduction, establishment and spread, or by enabling their prevention and management. Using Governance and Trade in this way enables us to assess how countries change their position in time in this fixed socio-economic space (see next section).

Consistent with the moderately strong positive correlation between Governance and Trade mentioned above (Figure S2), countries were roughly distributed within an elongated ellipse in the two-dimensional space (Fig. 3). Importantly, however, they were not evenly distributed across this ellipse. A cluster analysis revealed that countries can be grouped into four distinct clusters, roughly matching the four sectors defined by Governance and Trade (Figure S6; two widely used cluster algorithms, the complete linkage and the Ward algorithm, led to similar results, and we present results for the Ward algorithm hereafter). The cluster in the lower-left sector of the socio-economic space (negative Governance and Trade in our standardized scale) included 44 out of the 125 countries, of which 21 are from the set of 27 African countries used in our analyses. The cluster in the upper-right sector included 19 countries and is located further away from the other clusters in the socio-economic space than any other cluster. This category mostly included countries from the European Union (11 out of 25 countries) and some countries from other continents, such as Australia, New Zealand, USA, Canada, Japan and Singapore. The cluster located in the upper-left sector contained 49 (mostly Asian and Western European) countries. Finally, the cluster in the lower-right sector contained the smallest number of countries (13 countries). This cluster contained many island countries. Asian, South-American and African countries were spread over all four sectors, with Asian countries showing the highest variability in their distribution (Fig. 3).

### Temporal changes in predictors

**Fig. 4:**
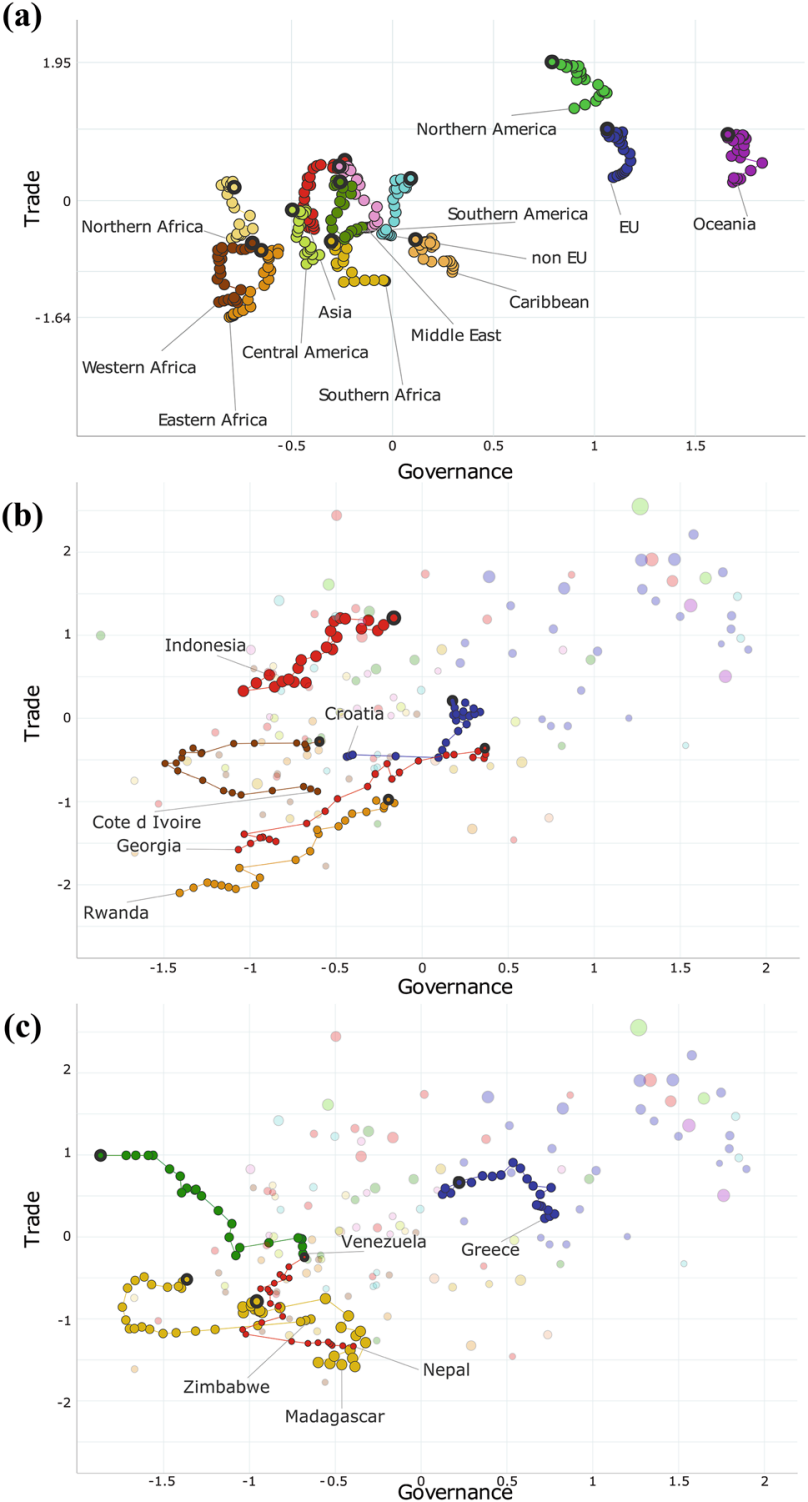
Changes in Governance and Trade between 1996 and 2018 for 125 countries. **a** Average changes for main geographic regions of the world. **b** Changes for countries with the largest increase of Governance. **c** Changes for countries with the largest decrease of Governance. Region and country names point towards positions in 1996, and thick bubbles represent positions in 2018. Bubble size illustrates EAS richness

Time lag phenomena are common in biological invasions, and our analyses showed that historical data better explained the recently observed EAS richness. To assess if countries are improving in their response capacity to EAS over time, we analysed the trajectories of countries in the two-dimensional socio-economic space defined by Governance and Trade during the past 20 years (Fig. 4). Increases in levels of Trade (or maintenance at high levels) should be correlated with EAS accumulation over time, whereas increases in levels of Governance should be correlated with a stagnation or even a decrease in EAS richness.

All countries have experienced an increase in level of Trade from 1996 to 2018, but changes in Governance were more variable. Countries from continents with high levels of economic development (Australasia, European Union [EU] and North America) demonstrated high levels of Governance (Fig. 4a). Their levels of Governance nonetheless tended to increase between 1996 and 2003, and then decreased until 2018. It was even lower in 2018 than in 1996 for North America (− 0.11 in our standardized scale for this predictor, with a maximum decline of − 0.27 between 2002 and 2018; Mexico, the USA and Canada showed qualitatively similar trajectories). Asian countries experienced the largest increase in their level of Governance on average (+ 0.18). Governance in North African countries has remained at a low level over this period. In contrast, West and East African countries started at a similar level as Northern African countries but saw the second and third largest increase in their level of Governance over time (+ 0.17 and + 0.16), especially after 2013 for West Africa. European countries that are not members of the European Union (non-EU) experienced a moderate increase (+ 0.10). Middle Eastern countries saw a rapid increase in the level of Governance between 1996 and 2000 (+ 0.32), with stable levels of Trade. After 2000, this trend reversed, with a stagnation in the level of Governance and an increase in the level of Trade. Middle Eastern, Caribbean, and especially Southern African countries saw the largest declines in their levels of Governance on average (− 0.13, − 0.18 and − 0.27, respectively). Results were much more heterogeneous at the country level, with some countries having large increases, decreases or fluctuations in their levels of Governance (Fig. 4b, c). Overall, countries with high levels of Governance in 1996 mostly remained close to their initial level. In contrast, countries with intermediate or low levels of Governance changed in either direction.

## Discussion

### Socio-economic and environmental predictors of biological invasions

Here, we provide a comprehensive quantitative analysis at the global scale of how countries perform in terms of a set of key socio-economic and environmental indices that are considered to be essential predictors of biological invasions and of the capacity of countries to mitigate their impacts (Essl et al. 2020b; Lenzner et al. 2020; Roura-Pascual et al. 2021). The relationships between some of the factors these indices characterise and alien species richness has been examined in other studies (using different sets of variables and indices), often over limited geographic regions (Vilà and Pujadas 2001; Pyšek et al. 2010; Essl et al. 2011; Seebens et al. 2015; Brenton-Rule et al. 2016; Early et al. 2016; Dawson et al. 2017a; Evans et al. 2018; Sardain et al. 2019). However, to our knowledge, no other study has examined the non-linear relationships between all these factors, EAS richness and country capacity to address biological invasions together over time at the global scale. Analyses limited to specific regions of the world such as Europe will only consider part of the socio-economic space (Fig. 3) and will therefore likely miss important relationships between socio-economic variables and biological invasions for lesser-studied regions of the world (Nuñez and Pauchard 2010). Broad-scale, correlative analyses based on general factors related to biological invasions are a necessary step to unveil complex interactions between these factors and start exploring future global scenarios of biological invasions in a quantitative fashion. This will pave the way towards more mechanistic models considering complex interactions between factors, and making the link with other factors more specific to biological invasion such as specific pathways, policies, or international agreements aimed at biological invasions (e.g. Early et al. 2016; Saul et al. 2017; Turbelin et al. 2017).

Although economic and environmental factors are often considered important and are well-understood, we show that societal, technological and especially political factors are also essential for obtaining a comprehensive perspective on spatial and temporal changes in biological invasions. As expected from other studies (Westphal et al. 2008; Banks et al. 2015; Seebens et al. 2015; Essl et al. 2020b; Lenzner et al. 2020; Hulme 2021), Trade was consistently the best predictor of EAS richness in one-predictor models, whereas the combination of Trade and Governance or Trade and Lifestyle and Education as main effects best explained EAS richness for most taxa in two-predictor models (although the decrease in AICc compared to models that only incorporated Trade was not always substantial). The factors characterized by these indices capture different aspects of biological invasions. Trade can facilitate the transportation of propagules and is therefore primarily and directly linked to the introduction stage of biological invasions (Blackburn et al. 2011). In contrast, Governance is related to all invasion stages, from introduction to establishment and spread of alien species, as it is a proxy for the capacity and willingness to design and implement adequate policies to prevent alien species from transiting from one stage to the other. As a proxy for these more direct factors, it was expected to show lower performance than a direct factor like Trade. Nonetheless, Governance appears to limit biological invasions at high levels only, whereas its correlation with EAS richness is positive at low values for most taxa, and even positive across the whole range of values for mammals (Fig. 1). Complex positive interactions between Governance and economic variables such as GDP or Trade levels have been shown in Eurasia (Evans et al. 2018), but our global-scale results suggest a different relationship. The non-linear relationship we observe likely reflects the complex interactions between Governance and other factors, including the fact that awareness and willingness to respond decisively to biological invasions may increase only once substantial negative impacts of IAS have been widely observed in a country. In addition, Governance is not independent from economic development overall, and was highly correlated with GNI. This may explain the positive correlation with EAS richness at low levels, where Governance may not be sufficiently advanced to counter the effects of economic developments on invasions such as land use change. The positive correlation between Governance and mammal EAS richness over the whole range of Governance levels may be due to the fact that annual alien mammal introductions have considerably decreased since 1950, contrary to other taxa (Seebens et al. 2017). As a result, patterns of mammal EAS richness will likely depend on index values much further in the past than what was available for our study.

Lifestyle and Education is another factor that proved to be important in our analyses, but has been largely neglected so far. Lifestyle and Education was the best predictor of EAS richness for ants when considering the 2015 data only. In combination with Trade, it also best explained EAS richness for ants, mammals and spiders even when considering historical data for Trade and Governance (Table S3). Lifestyle and Education was calculated by averaging the educational level of the population, the information globalization index and the cultural globalization index. Doing so enabled us to capture the potential level of understanding of complex issues such as biological invasions, but also connections with other cultures and countries, and the perception of nature (Table 1). Lifestyle and Education therefore has implications for alien species dispersal and establishment, e.g. via recreational activities and tourism, or mode of consumption. Importantly, Lifestyle and Education was also the best predictor for national reactive capacity and a good predictor for national proactive capacity. It is difficult at this stage to explain if this relationship is only correlative (e.g. countries investing in the education of their populations also tend to implement environmental policies), if there is a causal relationship (populations with high levels of education may vote for governments more inclined to design and implement environmental laws), or a combination of both. Our results nonetheless show that factors related to education and likely environmental awareness of a population, are important for predicting EAS richness and how countries will assess and react to the impacts caused by IAS. It is therefore essential to consider all aspects of country-level socio-economic and environmental changes to obtain a better perspective of potential future developments of biological invasions.

### Effects of historical legacies on recent levels of biological invasions

Our results underscore that invasion debt plays a crucial role in explaining recent levels of biological invasions (Essl et al. 2011). We found that historical data, where available (i.e. for Trade and Governance), overall better explained recent numbers of EAS than did recent data. Due to a lack of predictor data prior to 1996, we were not able to analyze if—and for how long—historical legacies extend beyond this time. Time lags also likely occur for the other factors, for which historical data were not available.

Biological invasions are affected by different factors at different stages of the invasion process (Rouget et al. 2016). For instance, while new alien species are introduced in response to changes in propagule pressure, introduced species become naturalized in response to human-induced changes in the recipient region and societal responses (e.g. IAS management, legislation) are adopted in response to observed or anticipated negative impacts (Brenton-Rule et al. 2016; Early et al. 2016; Turbelin et al. 2017). These processes may be associated with substantial lag times: detection of newly introduced species (Crooks 2005; Aikio et al. 2010) and their subsequent spread often occur after a time lag, which can delay the adoption and implementation of effective management (Pluess et al. 2012). Similarly, our findings show that historical levels of Governance, which are essential for the design and implementation of policies and the management of IAS, have an imprint on recent EAS richness in countries, an aspect that has been neglected in the literature so far. In particular, countries with higher levels of Governance 20 years ago tended to be less invaded than countries with intermediate Governance. Complex interactions between factors suggest that historical legacies may also apply to other factors. For example, since Lifestyle and Education was an important predictor for explaining proactive and reactive capacities of countries to address issues related to IAS (Figure S4), its relationship with EAS richness is likely to be subject to time lag. Past Lifestyle and Education may also be a good predictor of recent EAS richness, and it will likely be highly important for shaping future trajectories of EAS richness, as policies and management actions can take time to have effect. Historical legacies have been shown to extend far beyond 20 years for Trade (Essl et al. 2011), and it is possible that this also applies to the other indices. Historical legacies were nonetheless detected at different temporal scales (i.e. for 1996 or averaged over 1996–2015) for the different taxonomic groups. These differences may be due to several reasons. Different pathways of invasion are associated with different time-lags (Crooks 2005), and these variations may depend on species’ life cycles and dispersal potential. Meanwhile, the timing of sampling effort and the resulting data accuracy may be different between regions which may be invaded by different taxonomic groups. Finally, different taxonomic groups may have been considered differently by policy makers, and specific policies for different taxonomic groups and associated pathways of invasion may have been designed and implemented at different times under similar levels of Governance.

### A global picture of country positions in the socio-economic space through time and implications for alien species management and policies

Analyses of recent historical trajectories show that Trade has been increasing for all countries and will likely continue to do so in the next decades, with global freight demands predicted to increase three- to seven-fold between 2015 and 2050 (IMO 2015; OECD 2017). Recent research has shown that under a business-as-usual scenario, we can expect a global increase in EAS richness of 36% between 2005 and 2050 (Seebens et al. 2021). In the absence of effective biosecurity measures, the future intensification of Trade suggested by the past trajectories of most countries in the socio-economic space will inevitably be followed by large increases in species introductions. As a result, EAS richness may increase and largely exceed the business-as-usual estimations.

For Governance, recent historical trajectories are much less uniform across regions and countries. In particular, there are strong differences between different regions of the world, with increases for some regions, such as non-EU Europe and Asia, and declines for others, such as Central America and Southern Africa (Fig. 4). Differences are even larger at the country level and future country-specific projections for biological invasions, which are currently missing, would likely be highly uncertain. Overall, only high levels of Governance appear to have an effect on EAS richness (countries in the upper half or upper third bracket of levels of Governance, depending on the taxonomic group), probably because at low levels, Governance does not allow to counteract the effects of co-occurring economic development. Among geographic regions whose level of Governance increased between 1996 and 2018 (Fig. 4a), increases appear to be insufficient to reach the level of Governance at which it has an effect. Worse, the level of Governance of most geographic regions stagnated or even decreased over this period. Unless this trend is reversed, this will likely exacerbate the establishment of alien species whose rate of introduction will have also been raised by increases in Trade.

Our results show that countries strongly differ regarding essential socio-economic and environmental factors related to invasions. Causal relationships between the country-level indices used as predictors in our analyses and biological invasions are complex, and establishing causal links between these indices is beyond the scope of this publication. Nonetheless, it is important to note that all the factors we quantified in these analyses are related to different aspects of biological invasions, including their introduction, establishment, spread and management, and can therefore influence the future state of biological invasions (Essl et al. 2020b; Roura-Pascual et al. 2021). This implies that there are substantial opportunities for countries to mitigate the impacts of biological invasions in the future (e.g. identifying factors with the largest leverage or the potential to improve country ability to address biological invasions). Given the time lags involved in biological invasions, and the historical legacies of socio-economic and environmental factors on EAS richness, delays in positive changes, especially concerning Governance, may result in important long-term consequences for biodiversity.

Scenarios of biodiversity change that can inform decision-making are under development (Rosa et al. 2017; Leclère et al. 2020), but biological invasions are not considered in these analytical frameworks, despite the recognition of the importance of their integration into global environmental policies (e.g. Sustainable Development Goals; UN 2019). The on-going discussion on global targets for biodiversity conservation for the decades to come, including revised and specific targets on biological invasions (Essl et al. 2020a), highlights that integrating biological invasions into thematically broad assessments of environmental change is crucial. By revealing that large increases in levels of Governance are required to mitigate increases in EAS richness resulting from the expected intensification of Trade, and identifying the regions of the world where such changes are critically needed, our socio-economic space for biological invasions paves the way for such integration.

## Supplementary Information

Below is the link to the electronic supplementary material.

Supplementary file1 (DOCX 3235 KB)

Supplementary file2 (PDF 74 KB)

Supplementary file3 (CSV 100 KB)

## Data Availability

All data analyzed here are freely available from the original sources provided in Table 1. The data used as predictors for the three time periods (1996, 1996–2015, 2015) have been compiled in a single CSV file available in supplementary material.
