# Supplementary material for: Capacity of countries to reduce biological invasions
Source: Sustain Sci. 2022 Jul 20;18(2):771–89. doi: 10.1007/s11625-022-01166-3 (PMC10063504; doi:10.1007/s11625-022-01166-3)
Supplement: Supplementary file 1 — Supplementary file1 (DOCX 3235 KB) [file 11625_2022_1166_MOESM1_ESM.docx]

**Capacity of countries to reduce biological invasions**

**Supplementary Tables and Figures**

**Table S1.** Results of model selection for explaining EAS richness and national capacities in 125 countries based on the small-sample size corrected Akaike Information Criterion (AICc).

**Table S2.** Results of model selection for explaining EAS richness and national capacities in 125 countries based on the small-sample size corrected Akaike Information Criterion (AICc) using only the 2015 data.

**Table S3.** Results of model selection for explaining EAS richness and national capacities in 125 countries based on the small-sample size corrected Akaike Information Criterion (AICc) using only Governance and Trade, to compare recent and historical data.

**Figure S1.** geographic regions of the 125 countries (excluding some regions separate from mainland) included in the analyses. Countries in white were not considered due to data deficiency.

**Figure S2.** Relationships between five predictors and the respective variables (i.e. economy: Trade, policy: Governance, environment: Environmental Performance, social norms: Lifestyle and Education, technology: Innovation) of biological invasions used in the analyses.

**Figure S3.** Relationships between the five predictors and overall EAS richness.

**Figure S4.** Relationships between the five predictors (2015) and national capacities.

**Figure S5.** Cluster analysis of 125 countries in the socio-economic space based on Governance and Trade.

**Figure S6.** Moran’s *I* correlograms of residuals in the quadratic models for EAS richness.

**Figure S7.** Moran’s *I* correlograms of residuals in the quadratic models for national capacities to mitigate negative impacts of biological invasions.

**Table S1.** Results of model selection for explaining EAS richness and national capacities in 125 countries based on the small-sample size corrected Akaike Information Criterion (AICc) using only the 2015 data. Shown are the models with the lowest AICc values. ΔAICc_lin_ is the difference with the linear model with lowest AIC for 2015 (considering single- or two-predictor models). In italic are the polynomial models for which the lowest ΔAICc is not larger than 4 compared to the linear model including the same predictor. Underlined are the polynomial models for which the lowest ΔAICc is not larger than 4 compared to the best model including a single predictor. r^2^ values are the marginal variance.

| **Response variable** | **Predictors** | **Model type** | **ΔAICc_lin_** | **ΔAICc_one_** | **Marginal r^2^** |
| --- | --- | --- | --- | --- | --- |
| All taxa combined | Trade | Quadratic | -23.4 | 0 | 0.37 |
| Plants | Trade + Lifestyle & Education | Quadratic | -7.35 | -2.82  (Trade) | 0.51 |
| Ants | Trade + Lifestyle & Education | Quadratic | -16.43 | -8.73  (L&E) | 0.38 |
| Amphibians | Trade + Lifestyle & Education | Quadratic | -8.23 | -0.60  (Trade) | 0.54 |
| Reptiles | Trade + Environment | Cubic | -24.25 | -3.46  (Trade) | 0.46 |
| Fishes | Trade + Governance | Quadratic | -5.6 | -1.57  (Trade) | 0.53 |
| Birds | Trade + Lifestyle & Education | Quadratic | -10.7 | -5.84  (Trade) | 0.51 |
| Mammals | Trade + Lifestyle & Education | Quadratic | -6.99 | -4.3  (L&E) | 0.52 |
| Spiders | Trade + Lifestyle & Education | Quadratic | -12.96 | -8.08  (Trade) | 0.43 |
|  |  |  |  |  |  |
| Proactive national capacity | *Governance* | *Quadratic* | *-2.60* | *0* | *0.24* |
| Reactive national capacity | *Lifestyle & Education* | *Quadratic* | *-1.65* | *0* | *0.31* |

**Table S2.** Results of model selection for explaining EAS richness and national capacities in 125 countries based on the small-sample size corrected Akaike Information Criterion (AICc) using only Governance and Trade, to compare recent and historical data. Shown are the models with the lowest AICc values. ΔAICc_2015_ is the difference with the lowest 2015 values (considering single- or two-predictor models, but only Governance and Trade). ΔAICc_lin_ is the difference with the linear model with lowest AIC for all periods (considering single- or two-predictor models, but only Governance and Trade). ΔAICc_one_ is the difference AICc value with the models including only one of the two predictors. In italic are the polynomial models for which the lowest ΔAICc is not larger than 4 compared to the linear model including the same predictor. Underlined are the polynomial models for which the lowest ΔAICc is not larger than 4 compared to the best model including a single predictor. r^2^ values are the marginal variance.

| **Response variable** | **Predictors** | **Model type** | **Time period** | **ΔAICc_2015_** | **ΔAICc_lin_** | **ΔAICc_one_** | **Marginal r^2^** |
| --- | --- | --- | --- | --- | --- | --- | --- |
| All taxa combined | Trade | Cubic | 1996 | -24.37 | -39.85 | 0 | 0.39 |
| Plants | Trade | Quadratic | 1996-2015 | -2.08 | -6.12 | 0 | 0.48 |
| Ants | Trade + Governance | Quadratic | 1996-2015 | -1.89 | -12.72 | -8.47  (Trade) | 0.4 |
| Amphibians | Trade | Quadratic | 1996 | -8.46 | -10.27 | 0 | 0.56 |
| Reptiles | Trade + Governance | Cubic | 1996-2015 | -5.92 | -27.47 | -3.99  (Trade) | 0.44 |
| Fishes | Trade + Governance | Quadratic | 1996 | -5.86 | -6.58 | -2.32  (Trade) | 0.55 |
| Birds | Trade + Governance | Quadratic | 1996 | -19.76 | -13.26 | -5.67  (Trade) | 0.43 |
| Mammals | Trade + Governance | Quadratic | 1996 | -10.08 | -5.59 | -2.87  (Trade) | 0.49 |
| Spiders | Trade + Governance | Cubic | 1996 | -4.53 | -13.70 | -5.79  (Trade) | 0.31 |
|  |  |  |  |  |  |  |  |
| Proactive national capacity | Governance | *Quadratic* | *1996-2015* | *-1.05* | *-3.50* | *0* | *0.26* |
| Reactive national capacity | Trade | *Quadratic* | *1996* | *-2.59* | *-1.20* | *0* | *0.22* |

**Table S3.** Results of model selection for explaining EAS richness and national capacities in 125 countries based on the small-sample size corrected Akaike Information Criterion (AICc). Shown are the models with the lowest AICc values. ΔAICc_2015_ is the difference with the lowest 2015 values (considering single- or two-predictor models, for all predictors). ΔAICc_lin_ is the difference with the linear model with lowest AIC for all periods (considering single- or two-predictor models, for all predictors). In italic are the polynomial models for which the lowest ΔAICc is not larger than 4 compared to the linear model including the same predictor, and for which the linear model can therefore be considered as performing better. r^2^ values are the marginal variance.

| **Response variable** | **Predictors** | **Model type** | **Time period** | **ΔAICc_2015_** | **ΔAICc_lin_** | **Marginal r^2^** |
| --- | --- | --- | --- | --- | --- | --- |
| All taxa combined | Trade | Cubic | 1996 | -24.37 | -39.85 | 0.39 |
| Plants | Trade + Governance | Quadratic | 1996-2015 | 0.97 | -6.12 | 0.48 |
| Ants | Trade + Lifestyle & Education | Quadratic | 2015 | 0 | -15.67 | 0.38 |
| Amphibians | Trade | Quadratic | 1996 | -7.86 | -10.27 | 0.56 |
| Reptiles | Trade + Governance | Cubic | 1996-2015 | -5.77 | -27.47 | 0.44 |
| Fishes | Trade + Governance | Quadratic | 1996 | -5.86 | -6.58 | 0.55 |
| Birds | Trade + Governance | Quadratic | 1996 | -16.13 | -13.26 | 0.43 |
| Mammals | Trade + Lifestyle and Education | Quadratic | 2015 | 0 | -6.99 | 0.52 |
| Spiders | Trade + Lifestyle & Education | Quadratic | 2015 | 0 | -12.96 | 0.43 |
|  |  |  |  |  |  |  |
| Proactive national capacity | Governance | *Quadratic* | *1996-2015* | *-1.05* | *-3.50* | *0.26* |
| Reactive national capacity | Lifestyle & Education | *Quadratic* | *2015* | *0* | *-1.65* | *0.31* |


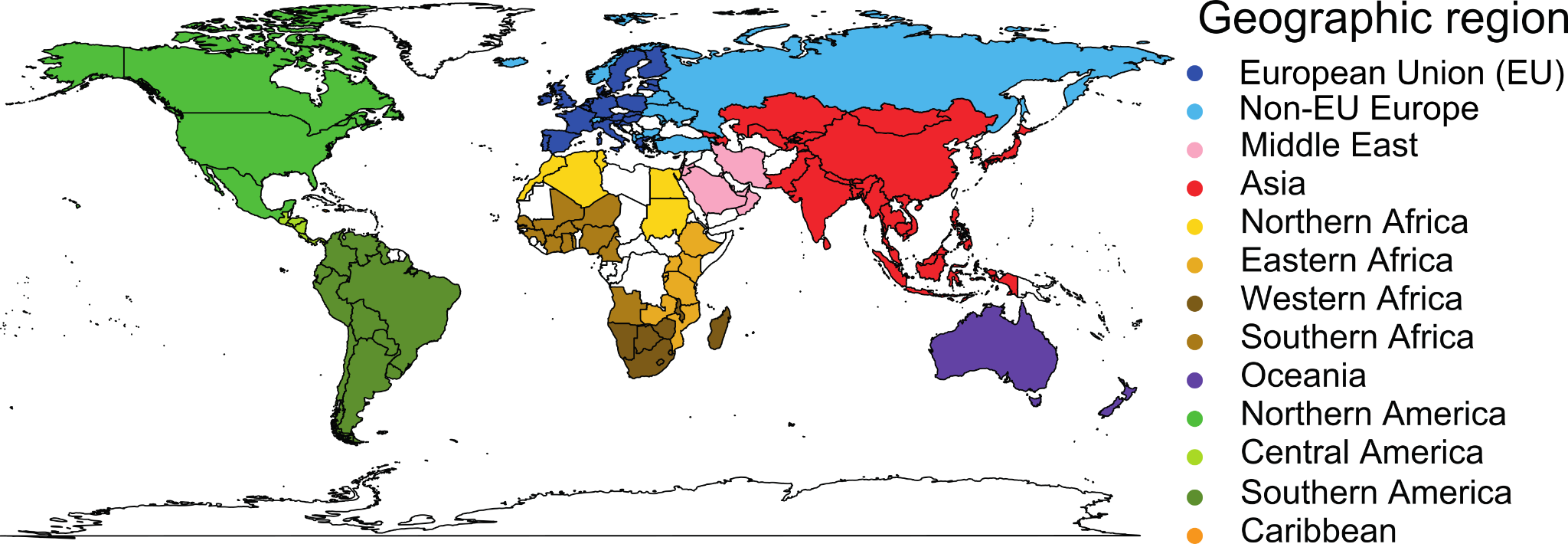


**Figure S1.** Geographic regions of the 125 countries (excluding overseas territories and territories separate from mainland) included in the analyses. Countries in white were not considered due to data deficiency.


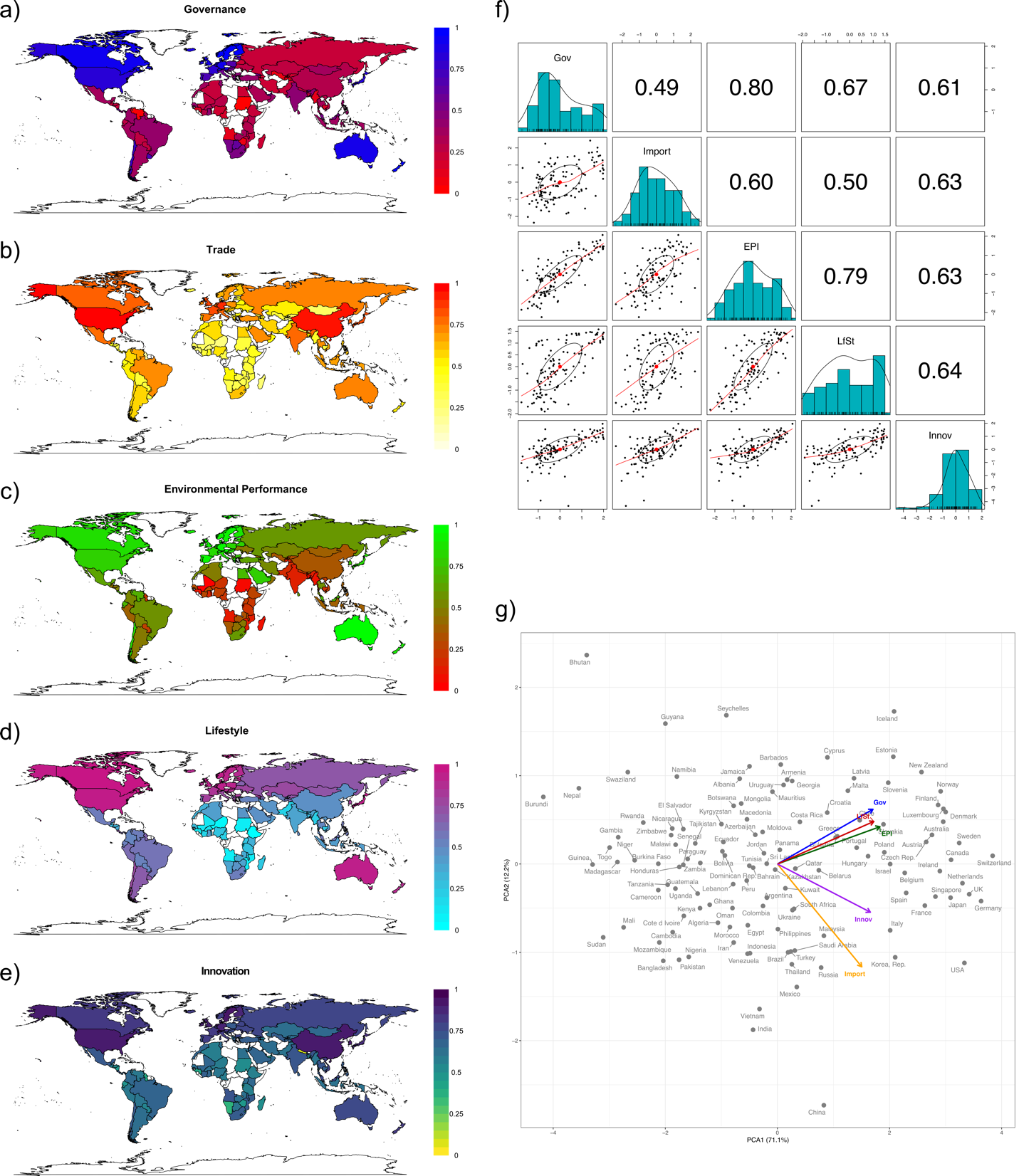


**Figure S2.** Relationships between the five indices (Trade, Governance, Environmental Performance, Lifestyle & Education, Innovation) considered important for biological invasions used in the analyses, after selecting appropriate variables and accounting for correlations between them (see section “Predictor selection and data” in the main text). a-e) The 125 countries (excluding some areas separate from mainland) used in the analyses are shown in different colors based on their levels for each of the five predictors. f) Pairwise Pearson's r correlation analyses between the indices. h) Principal component analyses of the five indices (blue: Governance, orange: Trade, green: Environmental Performance, red: Lifestyle and Education, purple: Innovation).


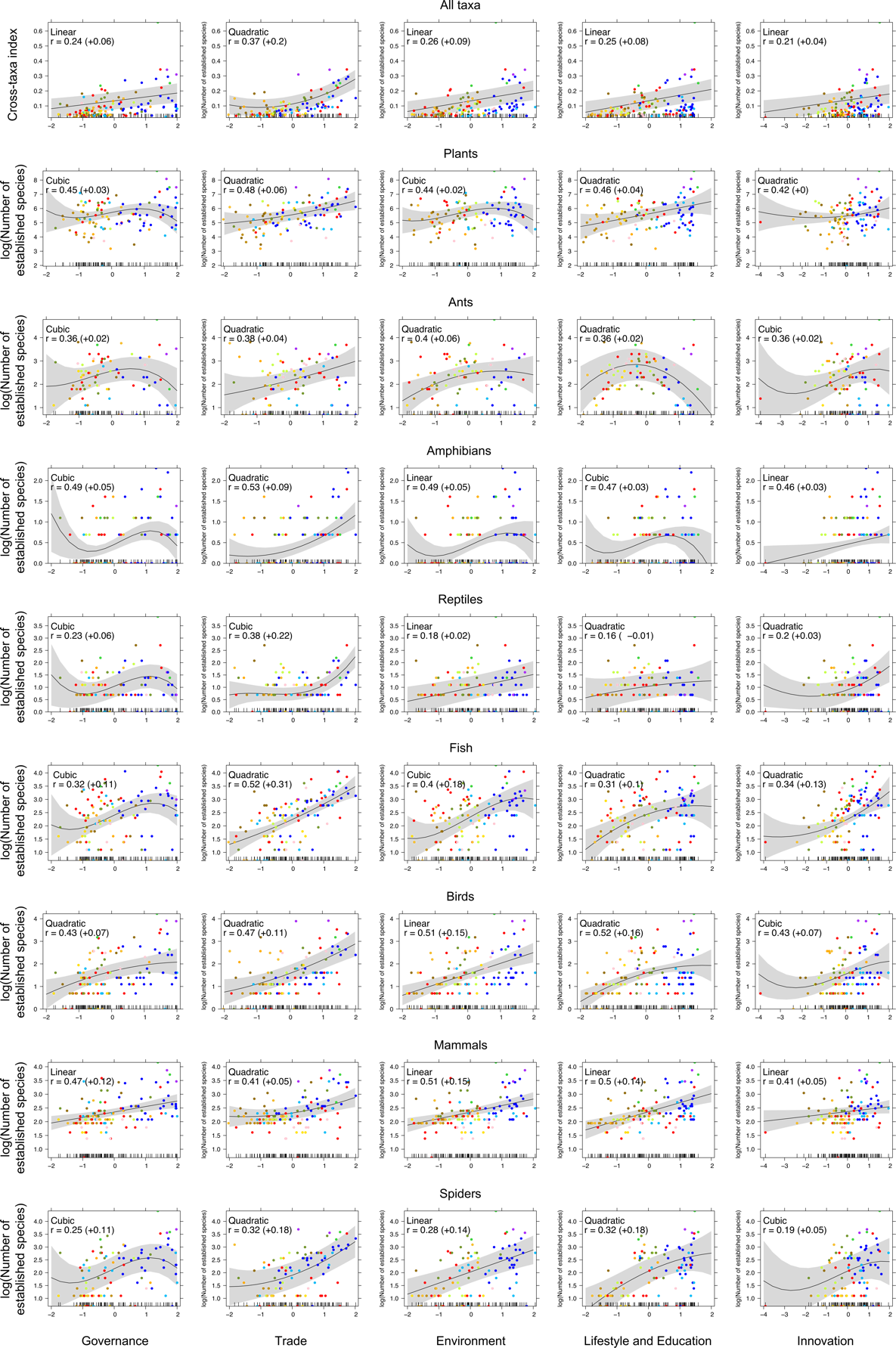


**Figure S3.** Relationships between the five predictors and overall EAS richness. The number of EAS was controlled for by country area, sampling effort, mean annual temperature and mean annual precipitation. The type of regression displayed is the one with the lowest AICc. The colors represent the geographic regions that the countries belong to (for legend, see Figures 1 and S1).


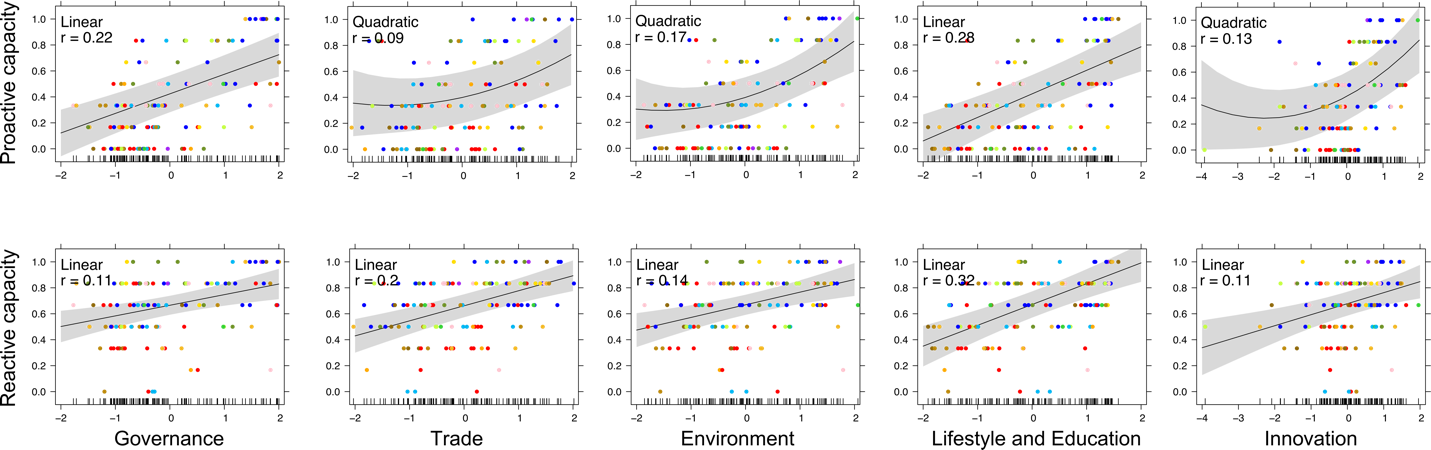


**Figure S4.** Relationships between the five predictors (2015) and national capacities. The type of regression displayed is the one with the lowest AICc. The colors represent the regions the geographic countries belong to (for legend, see Figures 1 and S1).


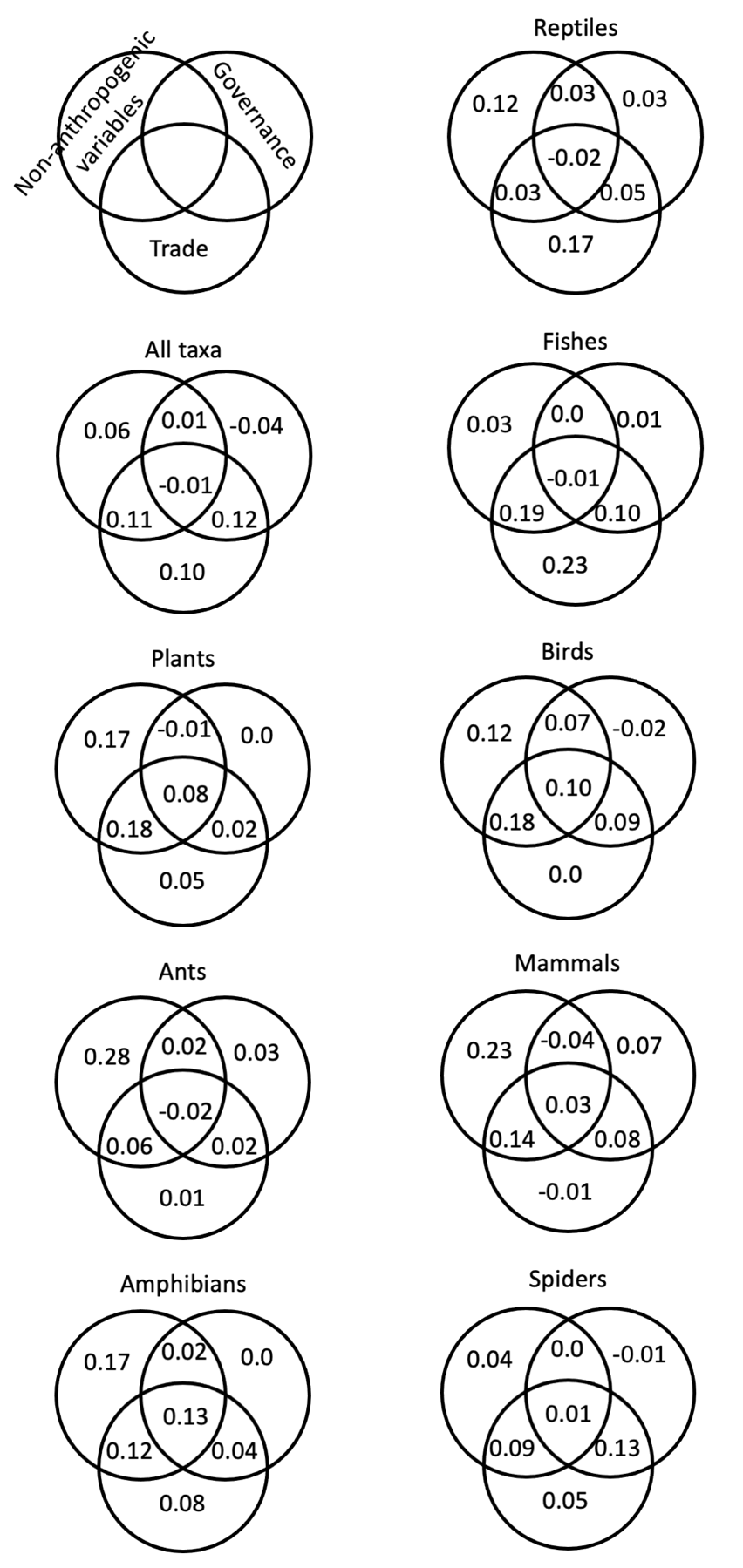


**Figure S5.** Variation partitioning assessing the respective contribution of the non-anthropogenic variables, Governance and Trade for the period generating the lowest AICc (Table S2) to EAS richness variation. Variation partitioning was computed based on marginal R^2^ values. Because changes in variables can change estimates of the random effect variance, that can lead to slightly negative values, and these results should be interpreted with caution.


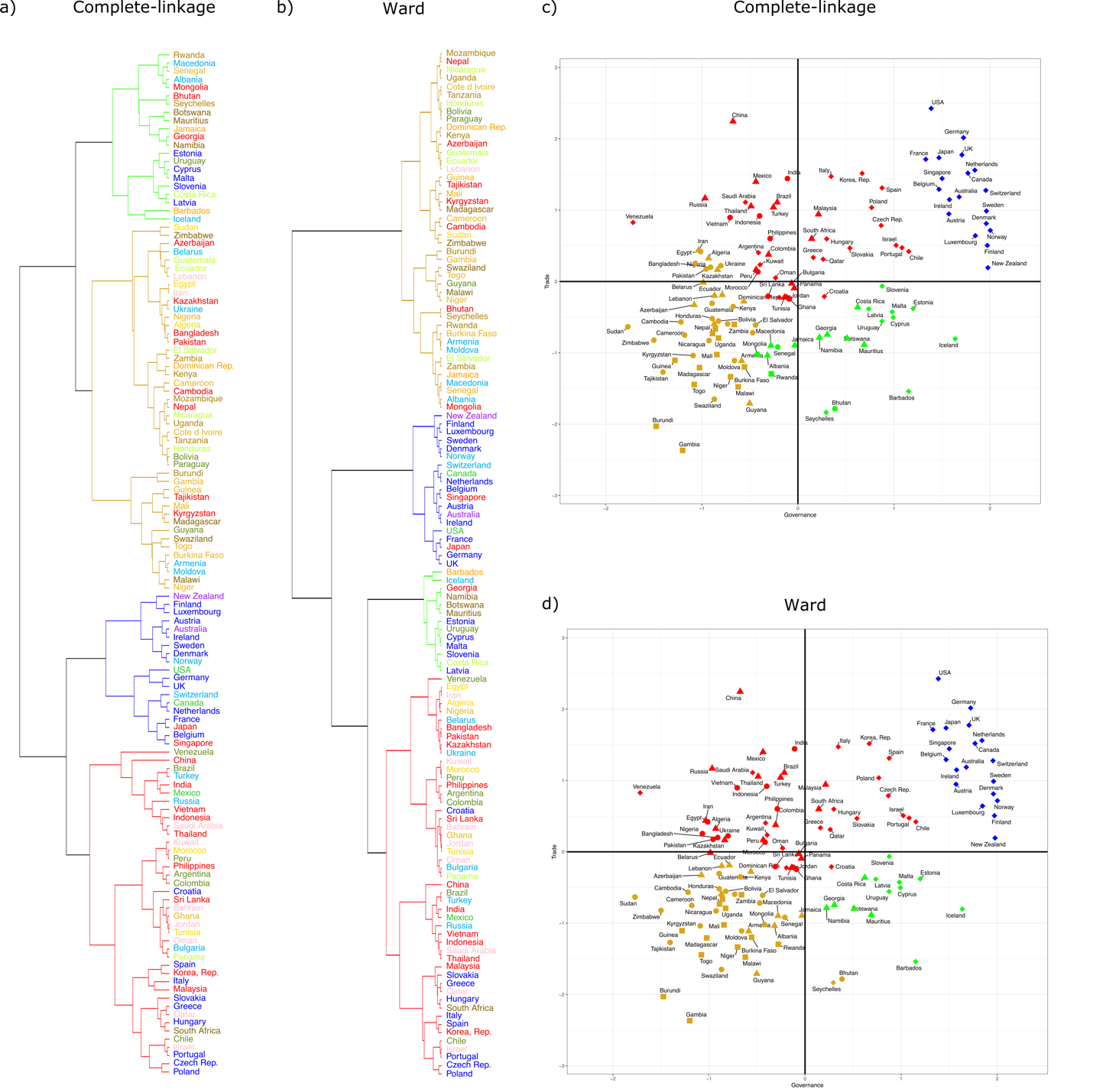


**Figure S6.** Cluster analysis of 125 countries in the socio-economic space based on Governance and Trade. a,b) Dendrograms based on country positions in the two-dimensional socio-economic space using two different cluster algorithms (i.e. complete-linkage and Ward). The colors of the country names represent the geographic regions (see Fig. S1). The colors of the branches represent the cluster countries belong to. c,d) Countries in the socio-economic space are colored according to the cluster they belong to.


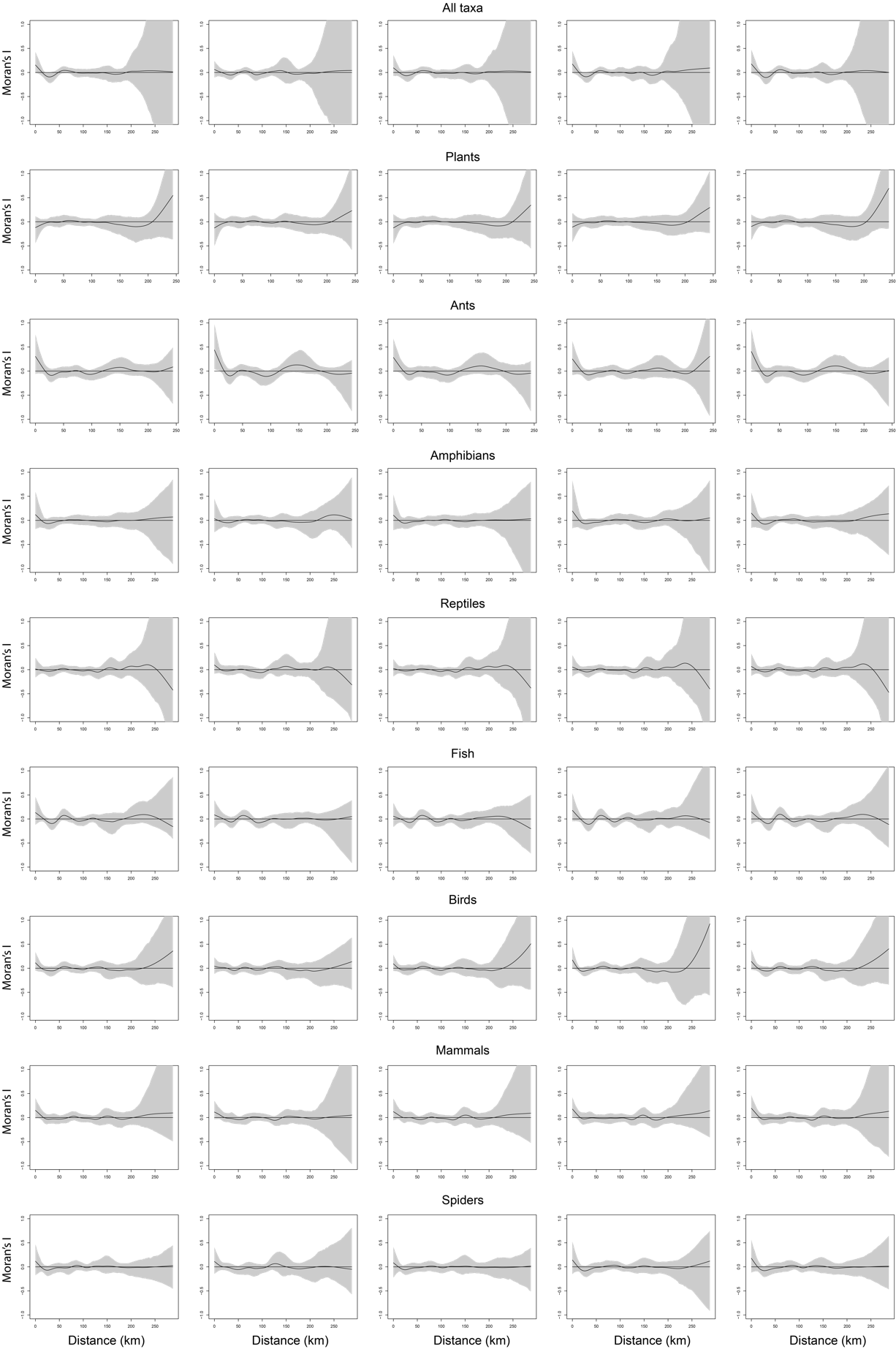


**Figure S7.** Moran’s *I* correlograms of residuals in the quadratic models for EAS richness. The X-axes show distance between country centroids. Spatial autocorrelation was always low at large and medium distances, and increases somewhat for some taxonomic groups at short distances, albeit not significantly.


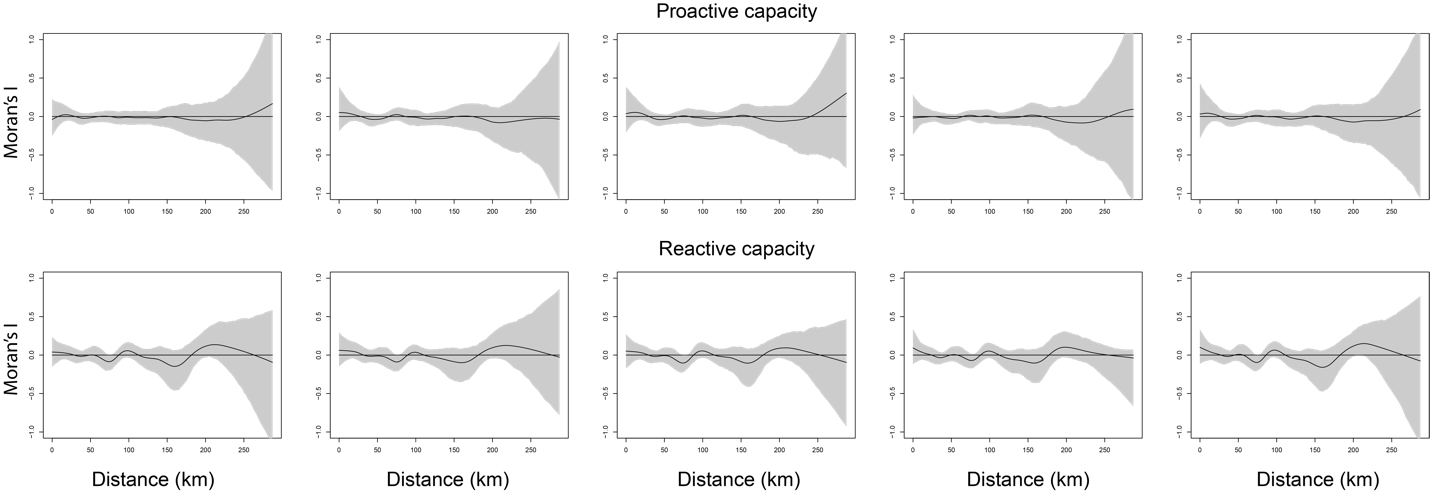


**Figure S8.** Moran’s *I* correlograms of residuals in the quadratic models for national capacities to mitigate negative impacts of biological invasions. The X-axes show distance between country centroids. Spatial autocorrelation was low in all models and at all distances.
