# Supplementary material for: Capacity of countries to reduce biological invasions
Source: Sustain Sci. 2022 Jul 20;18(2):771–89. doi: 10.1007/s11625-022-01166-3 (PMC10063504; doi:10.1007/s11625-022-01166-3)
Supplement: Supplementary file 2 — Supplementary file2 (PDF 74 KB) [file 11625_2022_1166_MOESM2_ESM.pdf]

Statistical analyses workflow

Data compilation

- Explore open access repositories with good transparency about the methods used to collate variables
- From these repositories, find country-scale variables related to the five main factors considered to be essential to understand and project future invasion dynamics: Governance, Trade, Environmental Performance, Lifestyle & Education, Innovation
- Extract environmental variables related to the biology and ecology of the species (here mean annual temperature and total annual precipitation), and to statistical aspects (country area and sampling effort)
- Extract country established alien species (EAS) richness from Dawnson et al. (2017) for the eight taxonomic groups, and aggregate the data across taxonomic groups to obtain an overall EAS richness index
- Extract the proactive and reactive capacity of countries to address biological invasions from Early et al. (2016)

Variable selection

- Discard variables that are more correlated with variables characterizing another factor than with variables characterizing the same factor (e.g. GNI characterizes Trade, but is more correlated with variables characterizing Governance, such as the Rule of Law, than with Imports in goods and services, and is therefore discarded).
- Average the remaining variables for each factor to create an index of Governance, Trade, Environmental Performance, Lifestyle & Education, Innovation.
- For Governance and Trade, compute the indices for 2015, 1996, and averaged over 1996-2015 (data not available for other factors).

Model regressions

- For each time period (2015, 1996, 1996-2015), compute a set of regression models to explain EAS richness and country capacities to address biological invasions
  - What is the shape of the relationship between predictor and response variables (linear, quadratic or cubic)?
  - Which period best explain the response variables?
- Base models (S = EAS richness [overall and for each taxonomic group], C = country capacity [proactive or reactive], A = area, E = sampling effort, T = temperature, P = precipitation, M = mainland/island, TWDG1 = geographic region):

$$S \sim A + E + A{\times}E + T + P + M + (1|TDWG1)$$
$$C \sim (1|TDWG1)$$

- One-variable models (X = Governance, Trade, Environmental Performance (2015 only), Lifestyle & Education (2015 only), or Innovation (2015 only)):

EAS richness (S)

$$S \sim X + A + E + A{\times}E + T + P + M + (1|TDWG1)$$
$$S \sim X + X^2 + A + E + A{\times}E + T + P + M + (1|TDWG1)$$
$$S \sim X + X^2 + X^3 + A + E + A{\times}E + T + P + M + (1|TDWG1)$$

Capacity (C)

$$C \sim X + (1|TDWG1)$$
$$C \sim X + X^2 + (1|TDWG1)$$
$$C \sim X + X^2 + X^3 + (1|TDWG1)$$

- Multi-variable models (X = Governance, Environmental Performance (2015 only) or Lifestyle & Education (2015 only), T = Trade):

EAS richness (S)

$$S \sim X + Tr + A + E + A{\times}E + T + P + M + (1|TDWG1)$$
$$S \sim X + G^2 + Tr + Tr^2 + A + E + A{\times}E + T + P + M + (1|TDWG1)$$
$$S \sim X + G^2 + G^3 + Tr + Tr^2 + Tr^3 + A + E + A{\times}E + T + P + M + (1|TDWG1)$$

Capacity (C)

$$C \sim X + Tr + (1|TDWG1)$$
$$C \sim X + G^2 + Tr + Tr^2 + (1|TDWG1)$$
$$C \sim X + G^2 + G^3 + Tr + Tr^2 + Tr^3 + (1|TDWG1)$$

Model selection and analysis

- Select models with the lowest AICc (ΔAICc > 4)
- Past Governance and Trade best explain EAS richness across taxonomic groups, and the proactive and reactive capacity of countries to address biological invasions
- EAS richness accelerated with Trade, but tends to decrease or slow down with Governance

Visualisation of socio-economic space

- Use Governance and Trade to define a two-dimensional space in which we can plot countries
- Explore how countries are distributed across this space provides a global perspective on how they differ in their capacity to address biological invasions
- Using space-for-time substitution, we can infer that countries should increase their level of governance through time to limit biological invasions, but many countries have not between 1996 and 2018
